# Assessment of the Allelochemical Activity and Biochemical Profile of Different Phenotypes of Picocyanobacteria from the Genus *Synechococcus*

**DOI:** 10.3390/md18040179

**Published:** 2020-03-27

**Authors:** Zofia Konarzewska, Sylwia Śliwińska-Wilczewska, Aldo Barreiro Felpeto, Vitor Vasconcelos, Adam Latała

**Affiliations:** 1 Division of Marine Ecosystems Functioning, Institute of Oceanography, University of Gdańsk, Av. Piłsudskiego 46, 81-378 Gdynia, Poland; ocessl@ug.edu.pl (S.Ś.-W.); oceal@ug.edu.pl (A.L.); 2 Interdisciplinary Center of Marine and Environmental Research–CIMAR/CIIMAR, University of Porto, Av. General Norton de Matos s/n, 4450-208 Matosinhos, Portugal; aldo.barreiro@gmail.com (A.B.F.); vmvascon@fc.up.pt (V.V.); 3 Department of Biology, Faculty of Sciences, Porto University, Rua do Campo Alegre, 4069-007 Porto, Portugal

**Keywords:** allelochemicals, phytochemicals, picocyanobacteria, *Synechococcus* sp.

## Abstract

Organisms belonging to *Synechococcus* sp. genera are observed in all freshwater, brackish, and marine waters of the world. They play a relevant role in these ecosystems, since they are one of the main primary producers, especially in open ocean. Eventually, they form mass blooms in coastal areas, which are potentially dangerous for the functioning of marine ecosystems. Allelopathy could be an important factor promoting the proliferation of these organisms. According to the authors’ best knowledge, there is no information on the allelopathic activity and allelopathic compounds exhibited by different *Synechococcus* sp. phenotypes. Therefore, the research conducted here aimed to study the bioactivity of compounds produced by three phenotypes of *Synechococcus* sp. by studying their influence on the growth, chlorophyll fluorescence, and photosynthetic pigments of eighteen cyanobacteria and microalgae species. We demonstrated that three different *Synechococcus* sp. phenotypes, including a phycocyanin (PC)-rich strain (Type 1; green strain) and phycoerythrin (PE)-rich strains containing phycoerythrobilin (PEB) and phycocyanobilin (PCB) (Type 2; red strain and Type 3a; brown strain), had a significant allelopathic effect on the selected species of cyanobacteria, diatoms, and green algae. For all green algae, a decrease in cell abundance under the influence of phenotypes of donor cyanobacteria was shown, whereas, among some target cyanobacteria and diatom species, the cell-free filtrate was observed to have a stimulatory effect. Our estimates of the stress on photosystem II (*F_v_/F_m_*) showed a similar pattern, although for some diatoms, there was an effect of stress on photosynthesis, while a stimulatory effect on growth was also displayed. The pigment content was affected by allelopathy in most cases, particularly for chlorophyll *a*, whilst it was a bit less significant for carotenoids. Our results showed that *Synechococcus* sp. Type 3a had the strongest effect on target species, while *Synechococcus* sp. Type 1 had the weakest allelopathic effect. Furthermore, GC-MS analysis produced different biochemical profiles for the *Synechococcus* strains. For every phenotype, the most abundant compound was different, with oxime-, methoxy-phenyl- being the most abundant substance for *Synechococcus* Type 1, eicosane for *Synechococcus* Type 2, and silanediol for *Synechococcus* Type 3a.

## 1. Introduction

Picoplanktonic cyanobacteria of the genus *Synechococcus* are of great importance for the functioning of marine ecosystems, due to their significance in the composition of phytoplankton communities [1]. Moreover, they are capable of producing harmful secondary metabolites [2], as well as creating blooms that play a significant role in the environment, which are enhanced as a result of the increasing eutrophication of coastal ecosystems [1,3] and global warming [4,5]. It is also possible that picocyanobacteria have an advantage over other organisms due to their ability to adapt to changing environmental conditions [2] and their allelopathic activity [6]. 

The dominant photosynthetic pigments (phycobiliproteins) in phycobilisomes constitute the basis of the classification of organisms from the genus *Synechococcus*, which is the most abundant picoplanktonic genus in the Baltic Sea [7]. Phycobilisomes of these picoplanktonic cyanobacteria can contain four different phycobiliproteins: phycocyanin (PC), two forms of phycoerythrin (PE) known as PEI and PEII, and allophycocyanin (APC). PC can tie phycoerythrobilin (PEB) and phycocyanobilin (PCB). PEI binds either only PEB or both PEB and PUB, whereas PEII always binds both PEB and PUB [8,9,10]. Furthermore, research conducted by Six et al. [9] created a classification of marine *Synechococcus* consisting of three large groups: Type 1 consists of phenotypes with PC; Type 2 consists of phenotypes with a dominance of PEI; and Type 3 incorporates phenotypes with PC and two types of PE, which can be subdivided into different types (from a to d) based on the PEB and PUB ratio, with Type 3a having a low PEB:PUB ratio [9]. All three phenotypes of picocyanobacteria occur in the Baltic Sea [11,12].

One of the reasons why allelopathy is a subject of interest for researchers is because it can favor the dominance of a species in the ecosystem [13]. However, the effect of allelochemicals depends on the nature of the interaction between donor and target organisms and the activity of the chemical compounds responsible for this interaction. Nevertheless, knowledge on the substances excreted by picoplanktonic cyanobacteria of the genus *Synechococcus* is still scarce. Recently, studies have been conducted on the allelopathic activity of a green strain of *Synechococcus*, with a higher amount of PC (Type 1) on phytoplankton species occurring in the same environment [2]. This indicates that these picocyanobacteria could constitute a source of allelochemical substances not previously identified. The aim of our work was to determine the bioactivity of compounds produced by a strain from each of the three phenotypes of *Synechococcus* sp. (Type 1, Type 2, and Type 3); the latter two of which have were been explored. The bioactivity was assessed by studying their influence on the growth, photosynthetic parameters, and pigment composition of coexisting phytoplanktonic species. In addition, a biochemical profile of each strain was obtained by GS-MS analysis.

## 2. Results and Discussion

### 2.1. Allelopathic Effect of Different Synechococcus sp. Phenotypes on the Growth of Targeted Species of Phytoplankton

**Figure 1:**
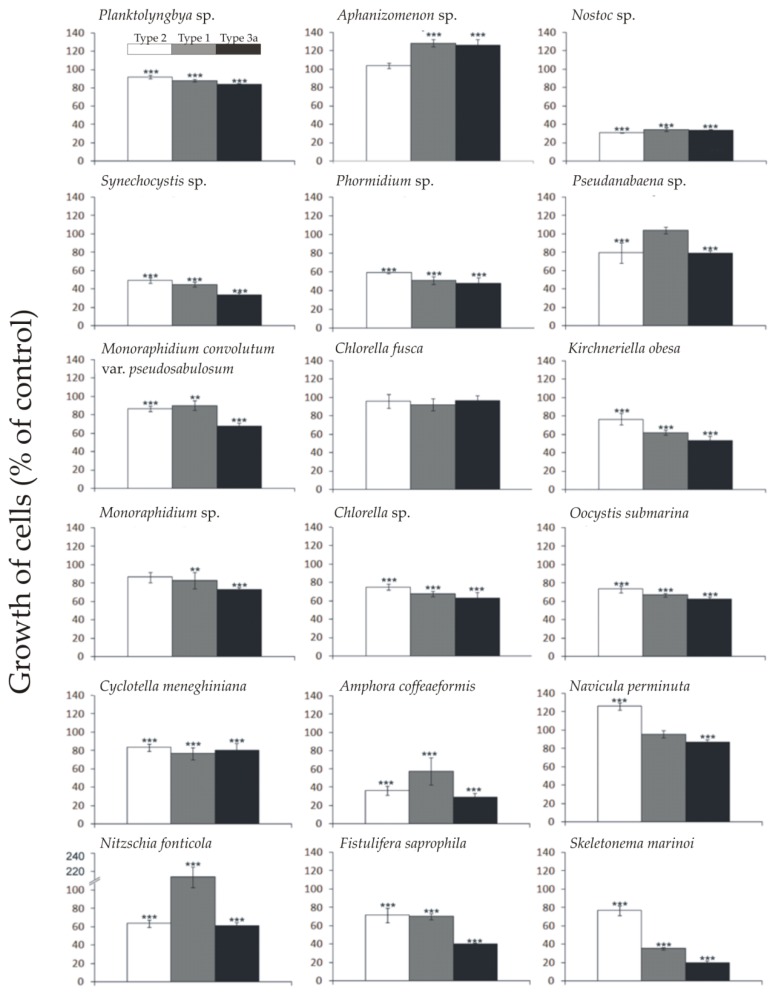
Growth of cells (% of control) of targeted cyanobacteria and microalgae, after the seventh day of the experiment with the addition of filtrate obtained from cultures of different *Synechococcus* sp. phenotypes. The values refer to means (*n* = 3, mean ± SD). Asterisk indicates significant difference identified by a post hoc Dunnett test, compared with the control (* *p* < 0.05; ** *p* < 0.01; *** *p* < 0.001).

Our results showed a significant effect of all three phenotypes of *Synechococcus* sp. on the growth of all target species of cyanobacteria and microalgae, except the green algae *Chlorella fusca* (Figure 1, Table S1 in Supplementary Materials). Experiments revealed that the filtrate obtained from donor picocyanobacteria, in the majority of cases, had a negative effect on the growth of target species, with the highest inhibition caused by *Synechococcus* Type 3a (brown strain) (Figure 1). The most significant allelopathic effect was observed for the diatom *Skeletonema marinoi* (ANOVA, *F*_9.32_ = 99.0; *p* < 0.001) and constituted 77% (Dunnett HSD, *p* < 0.001), 35% (Dunnett HSD, *p* < 0.001), and 20% (Dunnett HSD, *p* < 0.001), respectively, of growth observed in the control. Furthermore, the cyanobacterium for which the highest inhibition of growth was observed was *Nostoc* sp. (ANOVA, *F*_9.32_ = 283.1; *p* < 0.001) and constituted 31% (Dunnett HSD, *p* < 0.001), 34% (Dunnett HSD, *p* < 0.001), and 33% (Dunnett HSD, *p* < 0.001), respectively, compared to the control. A positive effect on growth was observed in one cyanobacterium, *Aphanizomenon* sp., by *Synechococcus* Type 1 (green strain) and Type 3a (brown strain), and diatoms species *Navicula perminuta* under the influence of *Synechococcus* Type 2 (red strain). The highest growth was noted for *Nitzschia fonticola* after the addition of cell-free filtrate of the green strain, which resulted in more than twice the number of cells of the control. 

In most cases, the highest allelopathic activity was demonstrated for *Synechococcus* Type 3a (brown strain). This phenotype belongs to cluster 5.1, which includes organisms that do not require an increased amount of nutrients [11]. Moreover, organisms in this clade prefer transparent ocean waters [14], and are thus not abundant in relatively nutrient-rich waters of the Baltic Sea. The strong growth-inhibiting response of the target phytoplankton strains to allelopathic compounds produced by the brown *Synechococcus* strain may have resulted from the geographical avoidance of both producer and target organisms and the consequent lack of adaptation. 

Several studies have shown a negative effect of the *Synechococcus* sp. Type 1 filtrates on different phytoplankton species, such as diatom *Navicula perminuta* [15], filamentous cyanobacteria *Nostoc* sp. and *Phormidium* sp. [16], bloom-forming cyanobacteria *Nodularia spumigena* [17], and *Porphyridium purpureum* and *Prymnesium parvum* [18]. Dominance by certain phytoplankton species in the community can be explained by the inhibition caused in the growth of co-existing species. If other environmental conditions are also favorable, this can lead to blooms of those species. This results in a change of the natural structure of the phytoplankton complex, for example, during the summer in the Baltic Sea [19,20].

It is believed that the plankton community maintains its diversity due to temporal (seasonal) heterogeneity driving them to a permanent non-equilibrium state, preventing species exclusion by a succession of seasonal communities [21]. Some factors contributing to the structuring of these seasonal communities have already been experimentally demonstrated, such as the light gradient [22]. However, allelopathy can also be considered as a driver of the plankton community structure [23]. Nevertheless, the effect of allelochemicals depends on the nature of the interaction between donor and target organisms and the activity of the chemical compounds responsible for this interaction.

In previous studies, it was demonstrated that allelopathic compounds may have self-stimulating properties [24]. Stimulation of the growth of the cyanobacteria *Aphanizomenon flos-aquae* under the influence of the filtrate from *Synechococcus* sp. was observed in studies conducted by Śliwińska-Wilczewska et al. [16]. Studies conducted by Bar-Yosef et al. [25] may be helpful in understanding the stimulating properties of allelopathic substances. Research has shown that *Aphanizomenon* can induce the alkaline phosphatase secretion of co-existing phytoplankton species, as a succession strategy. That is why an increase of target organisms is beneficial for bloom-forming *Aphanizomenon*.

### 2.2. Allelopathic Effect of Different Synechococcus sp. Phenotypes on the Chlorophyll Fluorescence of Studied Species of Phytoplankton

Our results showed significant effects of all three *Synechococcus* sp. phenotypes on the maximum PSII quantum efficiency (*F_v_/F_m_*) of the target species (Table 1, Table S2 in Supplementary Materials). The allelopathic effects on *F_v_/F_m_* were species-specific. Cell-free filtrate inhibited the *F_v_/F_m_* of all studied diatoms and green algae showed the strongest inhibition of the *F_v_/F_m_*, except *Oocystis submarina*, for which a positive effect was observed. However, cyanobacteria, in most cases, showed an increase in the *F_v_/F_m_*, suggesting a stimulatory effect of allelopathy. Among these, the highest increase was shown for *Phormidium* sp. (ANOVA, *F*_6.24_ = 15.9; *p* < 0.001) in response to *Synechococcus* Type 2 and constituted 158% of the control (Dunnett HSD, *p* < 0.001). However, the highest decrease in *F_v_/F_m_* was also observed for cyanobacterium. In *Nostoc* sp. (ANOVA, *F*_6.24_ = 11.7; *p* < 0.001), *F_v_/F_m_* dropped to only 29% of the control (Dunnett HSD, *p* < 0.001) after being exposed to *Synechococcus* Type 3a.

Prince et al. [26] showed a significant decrease in the *F_v_/F_m_* fluorescence parameter of the species *Akashiwo* cf. *sanguinea*, *Amphora* sp., *Asterionellopsis glacialis*, *Prorocentrum minimum*, and *Skeletonema costatum*in response to *Karenia brevis* (dinoflagellate). However, the studies conducted by Śliwińska-Wilczewska et al. [15] did not show a significant influence of diatom *Navicula perminuta* on the *F_v_/F_m_* value after the addition of filtrate obtained from *Synechococcus* sp. The filtrate from *Synechococcus* sp. also decreased the fluorescence parameter of *P. purpureum* and *Stichococcus bacillaris*, but increased the *F_v_/F_m_* of *P. parvum* [18]. Experiments conducted on freshwater species by Kovacs et al. [27] showed a significant decrease in the *F_v_/F_m_* of green alga *Scenedesmus quadricauda* after the addition of coexisting freshwater picocyanobacteria *Cyanobium gracile* and *Cylindrospermopsis raciborskii*. In addition, it was shown that the cell-free filtrate of picocyanobacteria *Synechocystis* sp. inhibits this same fluorescence parameter of the coexisting microalgae species *P. purpureum* and *Fistulifera* sp. [6].Our work and the mentioned literature show a predominantly inhibitory effect on this parameter. A low index of photosynthesis parameters is indicative of irregularities in the process of photosynthesis, low physiological conditions, and the impact of potential stress factors on the studied plant organisms [27]. Studies show that moderations in photosynthesis or the influence of chemicals alters fluorescence kinetics and may be an indicator of environmental changes [28]. A decrease of the main physiological process of co-occurring algae species is a competitor strategy, which can determine the domination of this species in the environment. The results showed that the influence of allelochemicals varied, depending on the strain, which may indicate that the composition of the phytoplankton community can heavily influence the nature of allelopathic interactions, as well as the seasonal succession [27].

### 2.3. Allelopathic Effect of Different Synechococcus sp. Phenotypes on the Photosynthetic Pigments of Studied Species of Phytoplankton

This study has shown that the filtrate from all phenotypes of *Synechococcus* sp. negatively affected the content of chlorophyll *a* and carotenoid pigments of the cyanobacteria *Phormidium* sp. and *Nostoc* sp., green algae *M. convolutum* var. *pseudosabulosum*, *K. obesa*, *Monoraphidium* sp., and *O. submarina*, as well as thediatoms *A. coffeaeformis*, *N. fonticola*, and *F. saprophila* (Table 2, Table S3 in Supplementary Materials). The largest decrease of chlorophyll *a* was found for the species *Monoraphidium* sp. with filtrate from phenotype 3a (6% relative to the control, *t*-test, *p* < 0.001) and the largest decrease of carotenoids was found for the species *M. convolutum var. pseudosabulosum* (16% relative to the control, *t*-test, *p* < 0.001). The green algae were shown to be the group most susceptible to allelopathic effects. The decrease in the content of pigments also appeared to be associated with the decrease in the number of cells of these organisms, with the exception of *N. fonticola*, which showed an increase in the number of cells under the influence of *Synechococcus* Type 1. Among the positive effects detected for the chlorophyll *a* content, the highest increase was demonstrated for cyanobacterium from the genus *Planktolyngbya*. There were no positive effects on carotenoid pigments observed. In general, carotenoids were much less affected than chlorophyll *a*.

In line with our results, Suikkanen et al. [20] observed a significant decrease in the chlorophyll *a* content of *Rhodomonas*sp. after being exposed to filtrate from cyanobacteria *A. flos-aquae* and *N. spumigena*. Additionally, a significant reduction of chlorophyll *a* in *Phormidium* sp., *Rivularia* sp., and *N. spumigena* was demonstrated in response to the filtrate from *Synechococcus* sp. [16,17]. Inhibition of the chlorophyll *a* value shows a drop in the efficiency of photosynthesis, which demonstrates the activity of the cell’s defense mechanism and response to stress factors [28,29]. 

### 2.4. GC-MS Analysis

**Figure 2:**
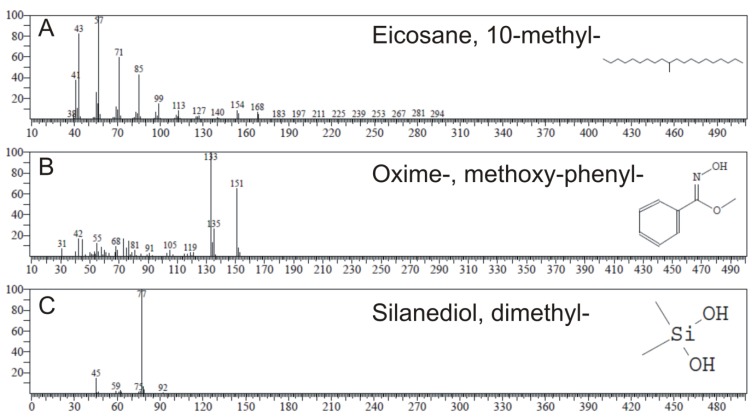
GC-MS spectrum of identified compounds which dominated in the cultures of different *Synechococcus* sp. phenotypes, including Type 2 (**A**), Type 1 (**B**), and Type 3a (**C**), and were not present in the f/2 medium.

GC-MS analysis identified the most abundant chemical compounds from every cell-free filtrate from *Synechococcus* sp. Type 1, Type 2, and Type 3a (Table 3) that did not occur in f/2 medium and thus may potentially be allelopathic substances. All substances present in the filtrates are shown in Table S4 in the Supplementary Materials, as well as the GC-MS chromatogram profiles of f/2 medium and three phenotypes of *Synechococcus* sp. in Figures S1–S4. Twenty-two chemical compounds not detected in the f/2 medium were found in *Synechococcus* Type 1 (green strain). The most abundant one was eicosane, 10-methyl- (Figure 2), with the peak area of 64.98%. *Synechococcus* Type 2 (red strain) showed 10 compounds that were not present in the f/2 medium. In this strain, the most abundant was oxime-, methoxy-phenyl-, with a peak area of 12.91%. GC-MS analysis revealed only five compounds produced by *Synechococcus* Type 3a (brown strain), with the most abundant of them being silanediol, dimethyl, with a 13.03% peak area. In previous studies, compounds detected in algae having antimicrobial properties included inter alia fatty acids, hydrocarbons, phenols, terpenes, and indoles [30,31,32], and some of these substances also occurred in the analysis carried out in this study. Chemicals from these classes were proven to have an antimicrobial and antialgal role, and can also be ranked among phytochemicals. 

Previous studies carried out using Gas Chromatography-Mass Spectrometry (GC-MS) analysis detected eicosane in species of red algae *Laurencia obtusa* var. *pyramidata* [33], brown algae *Turbinaria ornata* [34], and *Cystoseira barbata*, which was proven to show antimicrobial activity [35]. In addition, oxime-, methoxy-phenyl- found in *Synechococcus* Type 2 was identified in the bacteria *Sorangium cellulosum* [36] and *Pseudomonas aeruginosa* [37], fungi species *Aspergillus terreus* [38], and the leaf extract of *Alstonia scholaris* [39], and was proven to have antimicrobial, antibacterial, and antifungal properties. Furthermore, the most abundant substance in *Synechococcus* Type 3a was proven to be silanediol, which has protease inhibitor properties [40]. Moreover, studies conducted by Madsen et al. [41] have proven that silanediols represent a novel zinc binding group (ZBG) with properties that can be used for the development of histone deacetylase inhibitors, which have an important role inter alia in apoptosis [42].

Picocyanobacteria have also been rarely studied with respect to their potential as producers of novel bioactive allelopathic compounds. Our results indicate that different phenotypes of *Synechococcus* sp. may serve as a potential source of interesting bioactive compounds, whose characterization requires detailed investigation.

## 3. Materials and Methods

### 3.1. Studied Species

In this study, the experiments were conducted using *Synechococcus* strains from Type 1 (green strain), Type 2 (red strain), and Type 3a (brown strain), obtained from the Culture Collection of Baltic Algae (CCBA) as strains *Synechococcus* sp. (BA-120), *Synechococcus* sp. (BA-124), and *Synechococcus* sp. (BA-132), respectively. Eighteen species of cyanobacteria, green algae, and diatoms (Table 4) were cultivated in the CCBA at the Institute of Oceanography, University of Gdańsk, Poland [43], to be tested as target strains for investigation of the allelopathic activity of *Synechococcus*.

### 3.2. Culture Condition

Cyanobacterial and microalgal cultures were grown in f/2 medium [44]. Culture media was prepared with Baltic Sea water (salinity 8‰) filtered through glass fiber filters (Whatman GF/C) and autoclaved. All organisms were grown under the conditions of photosynthetic active light (PAR) of 10 μmol photons m^−2^s^−1^ with the photoperiod L:D 16:8, at 18 °C and 8‰. All cultures were acclimated to experimental conditions for 7 days. Light was provided by an artificial light source (Cool White 40W, Sylvania, Wilmington, MA, USA). Measurements of the PAR radiation intensity were made using the Li-Cor meter, model LI-189, with a cosine collector.

### 3.3. Determination of the Allelopathic Effect of Cell-Free Filtrates

Allelopathic effects were tested according to a method proposed by Suikkanen et al. [19], with modifications. Experimental treatments were prepared by adding 10 mL of the cell-free filtrate (f/2 medium in controls) to 25-mL Erlenmeyer flasks containing 10 mL of cell suspensions of the targeted species. In all experiments, the initial Chl *a* concentration in the cultures was 0.4 µg Chl *a* mL^−1^. *Synechococcus* sp. strains were filtered through a 0.45-µm filter (Macherey-Nagel MN GF-5). Tests were conducted in triplicate and the results of the experiments are presented as the mean value of three independent measurements.

### 3.4. Cell Density Assays

Cell abundances of cyanobacteria and microalgae were estimated with previously determined linear regression models on the basis of the optical density (OD) and number of cells (NmL^−1^) in the cultures. Estimates of the cell abundance of picocyanobacteria, green algae, and diatoms were carried out using the BD Accuri™ C6 Plus flow cytometer. Filamentous cyanobacteria cells were counted using a Tomic SFC-18 light microscope and the Bürker chamber, and were counted from 48 large squares [45]. Obtained data were used to fit a linear regression model of the number of cells and optical density (Table 5). Cell abundances were calculated on the seventh day of the exposure of target cells in experimental and control cultures. 

### 3.5. Fluorescence Assay

The effects of the filtrate of three different *Synechococcus sp*. phenotypes on the chlorophyll fluorescence of target species were determined using the maximum PSII quantum efficiency—*F_v_*/*F_m_* (where *F_v_*—the difference between the maximum and minimum fluorescence and *F_m_*—the maximum fluorescence) [46]. The measurements were conducted using a Pulse Amplitude Modulation (PAM) fluorometer (FMS1, Hansatech, King’s Lynn, United Kingdom) after 7 days of the experiment. Samples were filtered through glass fiber filters (Whatman GF/C, Saint Louis, MO, USA). Before measurement, the filtered sample was kept in the dark for approximately 10 min [15].

### 3.6. Pigments Assay

Photosynthetic pigments of target species were examined for the control and experiments with additions of the cell-free filtrate obtained from three different *Synechococcus* sp. phenotypes after 7 days of exposure. In the experiment, pigment extraction (4 mL of the material) was carried out in experimental flasks in 2 mL of 90% acetone in the dark and at a low temperature of −60 °C for about 1 hour. After this time, the extract was centrifuged for 1 min at 13,000 rpm min^−1^. Absorbance measurements were then carried out in 1 cm glass cuvettes on a Becker spectrophotometer model DU 530 at wavelengths of 480, 665, and 750 nm. For the determination of chlorophyll *a*, different formulas of cyanobacteria, green algae, and diatoms was used, as described by Jeffrey and Humphrey [47]. The concentration of carotenoid pigments was calculated using the formula employed by Strickland and Parsons [48].

### 3.7. GC–MS Analysis

GC-MS analysis was carried out on a Shimadzu QP 2017 SE LOG 149 system comprising a gas chromatograph interfaced to a mass spectrometer (GC-MS) instrument employing the following conditions: column Zebron 5MSi (30 m, I.D 0.25 mm × 0.25 µm, Phenomenex, Part no.: 7HG-G018-11, Serial nr. 357092), operating in electron impact mode at 70 eV; helium (99.999%) was used as carrier gas at a constant flow of 1.5 mL min^−1^ and an injection volume of 0.5 EI was employed (split ratio of 10:1) with an injector temperature of 300 °C; ion-source temperature 260 °C. The oven temperature was programmed from 30 °C (isothermal for 5 min), with an increase of 20 °C min^−1^, to 300 °C, ending with a 3 min isothermal at 300 °C. Mass spectra were taken at 70 eV, a scan-interval of 0.5 s, and fragments from 40 to 550 Da [49]. Compounds were identified by fitting coefficients (SI) using the NIST library ver. 14. 

### 3.8. Statistical Analyses

Repeated measures ANOVA was used to test the effect of *Synechococcus* strains on the growth and fluorescence of the targeted species onday0, 1, 3 (data not shown), and 7 of the experiment. A post hoc Dunnett test was used to determine significant differences between the control and the experimental treatments. One-way ANOVA was used to test the effect of picocyanobacterial filtrates on the chlorophyll *a* and carotenoid pigments in control and experimental cultures on the seventh day of the experiment. Data are reported as the mean ± standard deviation (SD). Levels of significance were * *p* < 0.05, ** *p* < 0.01, and *** *p* < 0.001. The statistical analyses were performed using the Statistica^®^ 13.1 software.

## 4. Conclusions

This work demonstrated the significant allelopathic effect of all three phenotypes of picoplanktonic cyanobacteria *Synechococcus* sp. on the growth and photosynthetic activity of selected species of Baltic cyanobacteria, green algae, and diatoms. GC-MS analysis of the cell-free filtrate showed the presence of chemical compounds that may potentially be allelopathic substances, of which the most dominant ones all had either antimicrobial or cell-damaging properties. Phenotypes of *Synechococcus* sp. displayed interesting and valuable allelopathic activities and further analysis of the substances they produce is essential, particularly due to the potential role of allelochemicals in structuring the plankton community.

## Figures and Tables

**Table 1:** Effect of filtrate from different *Synechococcus* sp. phenotypes on the chlorophyll fluorescence parameter *F_v_/F_m_* obtained after the seventh day of the experiment. Asterisk indicates significant difference identified by a post hoc Dunnett test, compared with the control (* *p* < 0.05; ** *p* < 0.01; *** *p* < 0.001).

Target Species	Effect on Phenotypes of Synechococcus sp.		
	Type 1	Type 2	Type 3a
**Cyanobacteria**			
*Planktolyngbya* sp.	+ *	0	0
*Aphanizomenon* sp.	+ **	+ ***	+ ***
*Nostoc* sp.	− ***	− ***	− ***
*Synechocystis* sp.	0	0	0
*Phormidium* sp.	+ ***	+ ***	+ ***
*Pseudanabaena* sp.	− ***	− ***	0
**Green algae**			
*Monoraphidium convolutum* var. *pseudosabulosum*	− ***	− ***	− ***
*Chlorella fusca*	− ***	− ***	− ***
*Kirchneriella obesa*	0	+ **	− **
*Monoraphidium* sp.	− ***	− ***	− ***
*Chlorella* sp.	− ***	− ***	− ***
*Oocystis* cf. *submarina*	+ ***	+ ***	+ ***
**Diatoms**			
*Cyclotella meneghiniana*	− *	− **	− *
*Amphora coffeaeformis*	− **	0	− ***
*Navicula perminuta*	− ***	0	0
*Nitzschia fonticola*	− ***	0	− ***
*Fistulifera saprophila*	− **	0	− ***
*Skeletonema marinoi*	0	0	− ***

**Table 2:** Effect of filtrate from different *Synechococcus* sp. phenotypes on photosynthetic pigments (chlorophyll *a* and carotenoids) obtained after the seventh day of the experiment. Asterisk indicates significant difference, compared with the control (* *p* < 0.05; ** *p* < 0.01; *** *p* < 0.001).

Target Species	Effect on Phenotypes of *Synechococcus* sp.					
	Type 1		Type 2		Type 3a	
	Photosynthetic Pigments					
	Chl *a*	Car	Chl *a*	Car	Chl *a*	Car
**Cyanobacteria**						
*Planktolyngbya* sp.	+ **	0	+ *	− *	+ **	0
*Aphanizomenon* sp.	+ *	0	0	0	+ **	0
*Nostoc* sp.	− *	− **	− *	− *	− *	− **
*Synechocystis* sp.	− *	0	− *	0	− *	0
*Phormidium* sp.	− ***	− ***	− ***	− ***	− ***	− *
*Pseudanabaena* sp.	− *	− ***	− *	0	− ***	− ***
**Green algae**						
*Monoraphidium convolutum* var. *pseudosabulosum*	− *	− **	− *	− **	− **	− ***
*Chlorella fusca*	− *	− *	0	0	− *	0
*Kirchneriella obesa*	− ***	− **	− ***	− **	− ***	− **
*Monoraphidium* sp.	− ***	− **	− ***	− **	− ***	− ***
*Chlorella* sp.	− ***	0	− ***	0	− ***	0
*Oocystis* cf. *submarina*	− ***	− **	− **	− **	− **	− **
**Diatoms**						
*Cyclotella meneghiniana*	− *	0	0	0	− ***	− **
*Amphora coffeaeformis*	− ***	− ***	− ***	− ***	− **	− ***
*Navicula perminuta*	− *	− *	0	0	− *	− *
*Nitzschia fonticola*	− ***	− **	− ***	− **	− ***	− ***
*Fistulifera saprophila*	− ***	− ***	− ***	− ***	− ***	− ***
*Skeletonema marinoi*	0	− **	0	0	− ***	− ***

**Table 3:** The most abundant phytochemicals identified in different phenotypes of picocyanobacteria from the genus *Synechococcus* Type 1 (green strain), Type 2 (red strain), and Type 3a (brown strain) by GC-MS.

Name of Compound	RT	Molecular	MW	Type 1		Type 2		Type 3a	
				Peak Area %	SI	Peak Area %	SI	Peak Area %	SI
Silanediol, dimethyl-	4.301	C_2_H_8_O_2_Si	92	ND	ND	ND	ND	13.03	97
Oxime-, methoxy-phenyl-	8.444	C_8_H_9_NO_2_	151	0.79	83	12.91	83	4.51	83
Eicosane, 10-methyl-	15.060	C_21_H_44_	296	64.98	96	6.73	94	ND	ND

RT = retention time, MW = molecular weight, ND = not detected, and SI = NIST (mass spectral libraries) Match Factors.

**Table 4:** Microalgae and cyanobacteria species tested for allelopathic activity.

Target Species	Identification in CCBA Collection
**Cyanobacteria**	
*Planktolyngbya* sp.	BA-50
*Aphanizomenon* sp.	BA-69
*Nostoc* sp.	BA-81
*Synechocystis* sp.	BA-121
*Phormidium* sp.	BA-141
*Pseudanabaena* sp.	BA-142
**Chlorophyta**	
*Monoraphidium convolutum* var. *pseudosabulosum*	BA-17
*Chlorella fusca*	BA-18
*Kirchneriella obesa*	BA-51
*Monoraphidium* sp.	BA-165
*Chlorella* sp.	BA-167
*Oocystis* cf. *submarina*	BA-172
**Bacilariophyta**	
*Cyclotella meneghiniana*	BA-10
*Amphora coffeaeformis*	BA-16
*Navicula perminuta*	BA-30
*Nitzschia fonticola*	BA-34
*Fistulifera saprophila*	BA-56
*Skeletonema marinoi*	BA-98

**Table 5:** Linear regression and correlation coefficients (*r*) used to calculate the number (*N*) of studied picocyanobacteria, cyanobacteria, green algae, and diatom cells in cultures based on optical density (OD) measurements.

Studied Strain	Linear Regression	Correlation Coefficient (*r*)
BA-120	*N* = 4242096·OD − 35834	0.97
BA-124	*N* = 93029379·OD − 98415	0.99
BA-132	*N* = 139120177·OD − 44353	0.99
BA-50	*N* = 74916153·OD + 46981	0.92
BA-69	*N* = 6716526·OD − 86633	0.96
BA-81	*N* = 39891877·OD − 11899	0.95
BA-121	*N* = 163917381·OD − 246275	0.98
BA-141	*N* = 86779699·OD − 44781	0.98
BA-142	*N* = 126415680·OD + 100972	0.98
BA-17	*N* = 24943668·OD − 263873	0.99
BA-18	*N* = 14395782·OD + 100101	0.97
BA-51	*N* = 12365968·OD − 246229	0.99
BA-165	*N* = 13120468·OD + 10489	0.99
BA-167	*N* = 3678299·OD + 274144	0.93
BA-172	*N* = 3363550·OD + 91273	0.98
BA-10	*N* = 8775538·OD − 1251	0.98
BA-16	*N* = 4385135·OD + 15527	0.98
BA-30	*N* = 6412449·OD − 8836	0.97
BA-34	*N* = 8050792·OD + 17824	0.98
BA-56	*N* = 7326981·OD − 57789	0.99
BA-98	*N* = 38103552·OD + 75013	0.97

where *N*—cells in 1 mL of medium and OD—optical density of the culture.

## Supplementary Materials

The following are available online at https://www.mdpi.com/1660-3397/18/4/179/s1, Figure S1: Chromatogram that shows the results of the analysis of sample of f/2 medium prepared using HS-SPME, Figure S2: Chromatogram that shows the results of the analysis of sample of phenotype Type 1 of *Synechococcus* sp. prepared using HS-SPME, Figure S3: Chromatogram that shows the results of the analysis of sample of phenotype Type 2 of *Synechococcus* sp. prepared using HS-SPME, Figure S4: Chromatogram that shows the results of the analysis of sample of phenotype Type 3a of *Synechococcus* sp. prepared using HS-SPME, Table S1:Number of cells of studied cyanobacteria and microalgae obtained after 7th day of the experiment for control and culture with the addition of filtrate obtained from cultures of cyanobacteria *Synechococcus* sp. (BA-120, BA-124 and BA-132), Table S2: Value of fluorescence of chlorophyll *a (F*_v_*/F*_m_ parameter) of studied cyanobacteria and microalgae obtained after 7th day of the experiment for control and culture with the addition of filtrate obtained from cultures of cyanobacteria *Synechococcus* sp. (BA-120, BA-124 and BA-132), Table S3: Value of photosynthetic pigments (chlorophyll *a* and carotenoid pigments) of studied cyanobacteria and microalgae obtained after 7th day of the experiment for control and culture with the addition of filtrate obtained from cultures of cyanobacteria *Synechococcus* sp. (BA-120, BA-124 and BA-132), Table S4: Phytochemicals identified in different phenotypes of picocyanobacteria from the genus *Synechococcus* Type 1 (green strain), Type 2 (red strain), and Type 3a (brown strain) by GC-MS. The compounds found in both the f/2 medium and picocyanobacterial cultures are highlighted in italics.
