# Supplementary material for: Assessment of the Allelochemical Activity and Biochemical Profile of Different Phenotypes of Picocyanobacteria from the Genus Synechococcus
Source: Mar Drugs. 2020 Mar 27;18(4):179. doi: 10.3390/md18040179 (PMC7230558; doi:10.3390/md18040179)
Supplement: Supplementary file 1 [file marinedrugs-18-00179-s001.pdf]

Supplementary material

Assessment of the Allelochemical Activity and Biochemical Profile of Different Phenotypes of Picocyanobacteria from the Genus *Synechococcus*

Zofia Konarzewska[1,*], Sylwia Śliwińska-Wilczewska[1], Aldo Barreiro Felpeto[2], Vitor Vasconcelos[2,3] and Adam Latała[1]

Table S1. Number of cells of studied cyanobacteria and microalgae obtained after 7th day of the experiment for control and culture with the addition of filtrate obtained from cultures of cyanobacteria *Synechococcus* sp. (BA-120, BA-124 and BA-132).

***Planktolyngbya* sp.**					
	Control$_1$	Control$_2$	Control$_3$	Mean	SD
OD (665 nm)	0.22	0.232	0.238	0.23	0.009165
OD (750 nm)	0.141	0.143	0.149	0.144333	0.004163
Number of cells (cell/ml)	10610159	10759991	11209488	10859879	311900.8
	BA-120$_1$	BA-120$_2$	BA-120$_3$	Mean	SD
OD (665 nm)	0.177	0.176	0.178	0.177	0.001
OD (750 nm)	0.133	0.131	0.134	0.132667	0.001528
Number of cells (cell/ml)	10010829	9860997	10085746	9985857	114436.3
% of control	94.35136	91.64503	89.97508	91.99049	2.208501
	BA-124$_1$	BA-124$_2$	BA-124$_3$	Mean	SD
OD (665 nm)	0.167	0.17	0.172	0.169667	0.002517
OD (750 nm)	0.125	0.127	0.129	0.127	0.002
Number of cells (cell/ml)	9411500	9561332	9711165	9561332	149832.3
% of control	88.70273	88.86004	86.63344	88.0654	1.242609
	BA-132$_1$	BA-132$_2$	BA-132$_3$	Mean	SD
OD (665 nm)	0.159	0.162	0.165	0.162	0.003
OD (750 nm)	0.119	0.12	0.124	0.121	0.002646
Number of cells (cell/ml)	8962003	9036919	9336584	9111836	198209.5
% of control	84.46625	83.98631	83.2918	83.91479	0.590484
Aphanizomenon sp.					
	Control$_1$	Control$_2$	Control$_3$	Mean	SD
OD (665 nm)	0.094	0.099	0.1	0.097667	0.003215
OD (750 nm)	0.065	0.068	0.069	0.067333	0.002082
Number of cells (cell/ml)	349941.2	370090.8	376807.3	365613.1	13981.56
	BA-120$_1$	BA-120$_2$	BA-120$_3$	Mean	SD
OD (665 nm)	0.096	0.1	0.107	0.101	0.005568
OD (750 nm)	0.065	0.071	0.072	0.069333	0.003786
Number of cells (cell/ml)	349941.2	390240.3	396956.9	379046.1	25428.36
% of control	100	105.4445	105.3474	103.5973	3.115744
	BA-124$_1$	BA-124$_2$	BA-124$_3$	Mean	SD
OD (665 nm)	0.12	0.126	0.131	0.125667	0.005508
OD (750 nm)	0.079	0.086	0.083	0.082667	0.003512
Number of cells (cell/ml)	443972.6	490988.2	470838.7	468599.8	23587.66
% of control	126.8706	132.667	124.9548	128.1641	4.015523
	BA-132$_1$	BA-132$_2$	BA-132$_3$	Mean	SD
OD (665 nm)	0.113	0.124	0.122	0.119667	0.005859
OD (750 nm)	0.076	0.086	0.083	0.081667	0.005132

Number of cells (cell/ml)	423823	490988.2	470838.7	461883.3	34466.53
% of control	121.1126	132.667	124.9538	126.2448	5.884205
***Nostoc* sp.**					
	$Control_1$	$Control_2$	$Control_3$	Mean	SD
OD (665 nm)	0.245	0.241	0.243	0.243	0.002
OD (750 nm)	0.17	0.165	0.166	0.167	0.002646
Number of cells (cell/ml)	6769720	6570261	6610153	6650044	105544
	BA-120_1	BA-120_2	BA-120_3	Mean	SD
OD (665 nm)	0.068	0.068	0.066	0.067333	0.001155
OD (750 nm)	0.052	0.052	0.051	0.051667	0.000577
Number of cells (cell/ml)	2062479	2062479	2022587	2049181	23031.59
% of control	30.46623	31.39112	30.59819	30.81851	0.500264
	BA-124_1	BA-124_2	BA-124_3	Mean	SD
OD (665 nm)	0.067	0.069	0.069	0.068333	0.001155
OD (750 nm)	0.055	0.059	0.059	0.057667	0.002309
Number of cells (cell/ml)	2182154	2341722	2341722	2288533	92126.34
% of control	32.23404	35.64123	35.42614	34.4338	1.908084
	BA-132_1	BA-132_2	BA-132_3	Mean	SD
OD (665 nm)	0.069	0.065	0.067	0.067	0.002
OD (750 nm)	0.058	0.053	0.056	0.055667	0.002517
Number of cells (cell/ml)	2301830	2102370	2222046	2208749	100392.4
% of control	34.00185	31.99828	33.61566	33.20526	1.062961
***Synechocystis* sp.**					
	$Control_1$	$Control_2$	$Control_3$	Mean	SD
OD (665 nm)	0.105	0.112	0.116	0.111	0.005568
OD (750 nm)	0.08	0.082	0.087	0.083	0.003606
Number of cells (cell/ml)	12867115	13194950	14014537	13358868	591012.5
	BA-120_1	BA-120_2	BA-120_3	Mean	SD
OD (665 nm)	0.06	0.057	0.059	0.058667	0.001528
OD (750 nm)	0.043	0.04	0.042	0.041667	0.001528
Number of cells (cell/ml)	6802172	6310420	6638255	6583616	250387.9
% of control	52.86478	47.82451	47.36692	49.35207	3.050687
	BA-124_1	BA-124_2	BA-124_3	Mean	SD
OD (665 nm)	0.055	0.053	0.056	0.054667	0.001528
OD (750 nm)	0.039	0.037	0.038	0.038	0.001
Number of cells (cell/ml)	6146503	5818668	5982585	5982585	163917.4
% of control	47.76908	44.09769	42.68843	44.85173	2.622918
	BA-132_1	BA-132_2	BA-132_3	Mean	SD
OD (665 nm)	0.043	0.04	0.042	0.041667	0.001528
OD (750 nm)	0.03	0.028	0.029	0.029	0.001
Number of cells (cell/ml)	4671246	4343412	4507329	4507329	163917.4
% of control	36.30376	32.91723	32.16181	33.79427	2.205862
***Phormidium* sp.**					
	$Control_1$	$Control_2$	$Control_3$	Mean	SD
OD (665 nm)	0.149	0.156	0.139	0.148	0.008544
OD (750 nm)	0.108	0.115	0.098	0.107	0.008544
Number of cells (cell/ml)	9327426	9934884	8459630	9240647	741446.1
	BA-120_1	BA-120_2	BA-120_3	Mean	SD
OD (665 nm)	0.071	0.083	0.065	0.073	0.009165
OD (750 nm)	0.063	0.071	0.057	0.063667	0.007024
Number of cells (cell/ml)	5422340	6116578	4901662	5480193	609520.6
% of control	58.13329	61.56667	57.9418	59.21392	2.039789

	BA-124$_1$	BA-124$_2$	BA-124$_3$	Mean	SD
OD (665 nm)	0.067	0.057	0.065	0.063	0.005292
OD (750 nm)	0.056	0.054	0.054	0.054667	0.001155
Number of cells (cell/ml)	4814882	4641323	4641323	4699176	100204.6
% of control	51.62069	46.71743	54.86437	51.0675	4.101547
	BA-132$_1$	BA-132$_2$	BA-132$_3$	Mean	SD
OD (665 nm)	0.066	0.059	0.054	0.059667	0.006028
OD (750 nm)	0.059	0.05	0.045	0.051333	0.007095
Number of cells (cell/ml)	5075221	4294204	3860305	4409910	615667.2
% of control	54.41181	43.22349	45.63209	47.75579	5.888735

***Pseudanabaena* sp.**

	Control$_1$	Control$_2$	Control$_3$	Mean	SD
OD (665 nm)	0.247	0.254	0.255	0.252	0.004359
OD (750 nm)	0.161	0.167	0.167	0.165	0.003464
Number of cells (cell/ml)	20453896	21212391	21212391	20959559	437916.8
	BA-120$_1$	BA-120$_2$	BA-120$_3$	Mean	SD
OD (665 nm)	0.212	0.206	0.2	0.206	0.006
OD (750 nm)	0.143	0.138	0.112	0.131	0.016643
Number of cells (cell/ml)	18178414	17546336	14259528	16661426	2103976
% of control	88.87507	82.71739	67.22264	79.60503	11.1567
	BA-124$_1$	BA-124$_2$	BA-124$_3$	Mean	SD
OD (665 nm)	0.255	0.252	0.25	0.252333	0.002517
OD (750 nm)	0.174	0.171	0.169	0.171333	0.002517
Number of cells (cell/ml)	22097300	21718053	21465222	21760192	318139.2
% of control	108.0347	102.3838	101.1919	103.8701	3.655508
	BA-132$_1$	BA-132$_2$	BA-132$_3$	Mean	SD
OD (665 nm)	0.168	0.161	0.175	0.168	0.007
OD (750 nm)	0.13	0.125	0.134	0.129667	0.004509
Number of cells (cell/ml)	16535010	15902932	17040673	16492872	570039.9
% of control	80.84039	74.97001	80.33358	78.71466	3.252847

Monoraphidium convolutum* var. *pseudosabulosum

	Control$_1$	Control$_2$	Control$_3$	Mean	SD
OD (665 nm)	0.423	0.469	0.476	0.456	0.028792
OD (750 nm)	0.335	0.373	0.38	0.362667	0.024214
Number of cells (cell/ml)	8092256	9040115	9214721	8782364	603994
	BA-120$_1$	BA-120$_2$	BA-120$_3$	Mean	SD
OD (665 nm)	0.362	0.379	0.391	0.377333	0.014572
OD (750 nm)	0.302	0.317	0.325	0.314667	0.011676
Number of cells (cell/ml)	7269115	7643270	7842819	7585068	291246.9
% of control	89.82804	84.54837	85.11185	86.49608	2.899279
	BA-124$_1$	BA-124$_2$	BA-124$_3$	Mean	SD
OD (665 nm)	0.379	0.38	0.405	0.388	0.014731
OD (750 nm)	0.322	0.32	0.342	0.328	0.012166
Number of cells (cell/ml)	7767988	7718101	8266861	7917650	303452.8
% of control	95.99286	85.37613	89.71364	90.36088	5.337877
	BA-132$_1$	BA-132$_2$	BA-132$_3$	Mean	SD
OD (665 nm)	0.274	0.278	0.287	0.279667	0.006658
OD (750 nm)	0.244	0.246	0.254	0.248	0.005292
Number of cells (cell/ml)	5822382	5872269	6071819	5922157	131989.5

% of control	71.95005	64.9579	65.89259	67.60018	3.795973
Chlorella fusca					
	$Control_1$	$Control_2$	$Control_3$	Mean	SD
OD (665 nm)	0.27	0.302	0.281	0.284333	0.016258
OD (750 nm)	0.22	0.245	0.23	0.231667	0.012583
Number of cells (cell/ml)	3267173	3627068	3411131	3435124	181143
	$BA\text{-}120_1$	$BA\text{-}120_2$	$BA\text{-}120_3$	Mean	SD
OD (665 nm)	0.275	0.26	0.269	0.268	0.00755
OD (750 nm)	0.226	0.214	0.223	0.221	0.006245
Number of cells (cell/ml)	3353548	3180798	3310360	3281569	89901.63
% of control	102.6437	87.69614	97.04583	95.79523	7.551853
	$BA\text{-}124_1$	$BA\text{-}124_2$	$BA\text{-}124_3$	Mean	SD
OD (665 nm)	0.254	0.251	0.269	0.258	0.009644
OD (750 nm)	0.209	0.207	0.222	0.212667	0.008145
Number of cells (cell/ml)	3108819	3080028	3295965	3161604	117246.8
% of control	95.15319	84.91785	96.62381	92.23162	6.376446
	$BA\text{-}132_1$	$BA\text{-}132_2$	$BA\text{-}132_3$	Mean	SD
OD (665 nm)	0.271	0.281	0.253	0.268333	0.014189
OD (750 nm)	0.226	0.233	0.211	0.223333	0.01124
Number of cells (cell/ml)	3353548	3454318	3137611	3315159	161805.9
% of control	102.6437	95.23722	91.98155	96.62083	5.464085
Kirchneriella obesa					
	$Control_1$	$Control_2$	$Control_3$	Mean	SD
OD (665 nm)	0.496	0.527	0.542	0.521667	0.023459
OD (750 nm)	0.404	0.433	0.451	0.429333	0.023714
Number of cells (cell/ml)	4749622	5108235	5330823	5062893	293241.2
	$BA\text{-}120_1$	$BA\text{-}120_2$	$BA\text{-}120_3$	Mean	SD
OD (665 nm)	0.381	0.356	0.366	0.367667	0.012583
OD (750 nm)	0.341	0.326	0.33	0.332333	0.007767
Number of cells (cell/ml)	3970566	3785077	3834540	3863394	96052.08
% of control	83.59752	74.09754	71.9315	76.54218	6.205338
	$BA\text{-}124_1$	$BA\text{-}124_2$	$BA\text{-}124_3$	Mean	SD
OD (665 nm)	0.294	0.294	0.308	0.298667	0.008083
OD (750 nm)	0.269	0.269	0.283	0.273667	0.008083
Number of cells (cell/ml)	3080216	3080216	3253340	3137924	99952.93
% of control	64.85182	60.29903	61.02885	62.0599	2.445253
	$BA\text{-}132_1$	$BA\text{-}132_2$	$BA\text{-}132_3$	Mean	SD
OD (665 nm)	0.234	0.248	0.297	0.259667	0.033081
OD (750 nm)	0.214	0.226	0.272	0.237333	0.030616
Number of cells (cell/ml)	2400088	2548480	3117314	2688627	378595.2
% of control	50.53219	49.88963	58.47717	52.96633	4.783329
***Monoraphidium* sp.**					
	$Control_1$	$Control_2$	$Control_3$	Mean	SD
OD (665 nm)	0.416	0.464	0.436	0.438667	0.024111
OD (750 nm)	0.33	0.374	0.349	0.351	0.022068
Number of cells (cell/ml)	4340243	4917544	4589532	4615773	289543.5
	$BA\text{-}120_1$	$BA\text{-}120_2$	$BA\text{-}120_3$	Mean	SD
OD (665 nm)	0.355	0.361	0.38	0.365333	0.013051
OD (750 nm)	0.294	0.299	0.315	0.302667	0.01097
Number of cells (cell/ml)	3867907	3933509	4143436	3981617	143927
% of control	89.11727	79.9893	90.28014	86.46224	5.635804
	$BA\text{-}124_1$	$BA\text{-}124_2$	$BA\text{-}124_3$	Mean	SD

OD (665 nm)	0.36	0.329	0.358	0.349	0.017349
OD (750 nm)	0.3	0.273	0.297	0.29	0.014799
Number of cells (cell/ml)	3946629	3592377	3907268	3815425	194165.2
% of control	90.93106	73.05225	85.13434	83.03922	9.121682
	BA-132$_1$	BA-132$_2$	BA-132$_3$	Mean	SD
OD (665 nm)	0.29	0.306	0.292	0.296	0.008718
OD (750 nm)	0.25	0.262	0.252	0.254667	0.006429
Number of cells (cell/ml)	3290606	3448052	3316847	3351835	84352.81
% of control	75.81616	70.11735	72.26982	72.73445	2.877676
**Chl*orella* sp.**					
	Control$_1$	Control$_2$	Control$_3$	Mean	SD
OD (665 nm)	0.457	0.529	0.507	0.497667	0.036896
OD (750 nm)	0.389	0.451	0.431	0.423667	0.031644
Number of cells (cell/ml)	1705002	1933057	1859491	1832517	116395.5
	BA-120$_1$	BA-120$_2$	BA-120$_3$	Mean	SD
OD (665 nm)	0.312	0.328	0.324	0.321333	0.008327
OD (750 nm)	0.289	0.304	0.3	0.297667	0.007767
Number of cells (cell/ml)	1337172	1392347	1377634	1369051	28571.02
% of control	78.42643	72.02824	74.08661	74.84709	3.266182
	BA-124$_1$	BA-124$_2$	BA-124$_3$	Mean	SD
OD (665 nm)	0.268	0.285	0.282	0.278333	0.009074
OD (750 nm)	0.252	0.267	0.265	0.261333	0.008145
Number of cells (cell/ml)	1201075	1256250	1248893	1235406	29958.01
% of control	70.44421	64.98773	67.16318	67.53171	2.74684
	BA-132$_1$	BA-132$_2$	BA-132$_3$	Mean	SD
OD (665 nm)	0.263	0.242	0.256	0.253667	0.010693
OD (750 nm)	0.247	0.227	0.241	0.238333	0.010263
Number of cells (cell/ml)	1182684	1109118	1160614	1150805	37751.13
% of control	69.36553	57.37637	62.41569	63.05253	6.019896
Oocystis* cf. *submarina					
	Control$_1$	Control$_2$	Control$_3$	Mean	SD
OD (665 nm)	0.327	0.328	0.344	0.333	0.009539
OD (750 nm)	0.27	0.27	0.286	0.275333	0.009238
Number of cells (cell/ml)	999431.5	999431.5	1053248	1017370	31071.14
	BA-120$_1$	BA-120$_2$	BA-120$_3$	Mean	SD
OD (665 nm)	0.218	0.229	0.214	0.220333	0.007767
OD (750 nm)	0.192	0.201	0.19	0.194333	0.005859
Number of cells (cell/ml)	737074.6	767346.6	730347.5	744922.9	19708.6
% of control	73.74939	76.7783	69.34239	73.29003	3.739181
	BA-124$_1$	BA-124$_2$	BA-124$_3$	Mean	SD
OD (665 nm)	0.187	0.201	0.2	0.196	0.00781
OD (750 nm)	0.168	0.179	0.18	0.175667	0.006658
Number of cells (cell/ml)	656349.4	693348.5	696712	682136.6	22395.62
% of control	65.67227	69.37428	66.14888	67.06515	2.01392
	BA-132$_1$	BA-132$_2$	BA-132$_3$	Mean	SD
OD (665 nm)	0.188	0.179	0.181	0.182667	0.004726
OD (750 nm)	0.167	0.158	0.162	0.162333	0.004509
Number of cells (cell/ml)	652985.9	622713.9	636168.1	637289.3	15167.09
% of control	65.33573	62.30681	60.40058	62.68104	2.488765
Cyclotella meneghiniana					
	Control$_1$	Control$_2$	Control$_3$	Mean	SD
OD (665 nm)	0.096	0.111	0.11	0.105667	0.008386

OD (750 nm)	0.062	0.071	0.07	0.067667	0.004933
Number of cells (cell/ml)	542832.4	621812.2	613036.7	592560.4	43288.7
	BA-120$_1$	BA-120$_2$	BA-120$_3$	Mean	SD
OD (665 nm)	0.078	0.081	0.084	0.081	0.003
OD (750 nm)	0.052	0.056	0.061	0.056333	0.004509
Number of cells (cell/ml)	455077	490179.1	534056.8	493104.3	39571.09
% of control	83.8338	78.83074	87.11662	83.26038	4.172598
	BA-124$_1$	BA-124$_2$	BA-124$_3$	Mean	SD
OD (665 nm)	0.074	0.074	0.072	0.073333	0.001155
OD (750 nm)	0.052	0.051	0.052	0.051667	0.000577
Number of cells (cell/ml)	455077	446301.4	455077	452151.8	5066.559
% of control	83.8338	71.77431	74.23324	76.61378	6.372443
	BA-132$_1$	BA-132$_2$	BA-132$_3$	Mean	SD
OD (665 nm)	0.076	0.074	0.074	0.074667	0.001155
OD (750 nm)	0.055	0.053	0.054	0.054	0.001
Number of cells (cell/ml)	481403.6	463852.5	472628.1	472628.1	8775.538
% of control	88.68366	74.59688	77.09621	80.12558	7.516125
Amphora coffeaeformis					
	Control$_1$	Control$_2$	Control$_3$	Mean	SD
OD (665 nm)	0.037	0.033	0.038	0.036	0.002646
OD (750 nm)	0.024	0.02	0.024	0.022667	0.002309
Number of cells (cell/ml)	120770.2	103229.7	120770.2	114923.4	10127.04
	BA-120$_1$	BA-120$_2$	BA-120$_3$	Mean	SD
OD (665 nm)	0.014	0.013	0.012	0.013	0.001
OD (750 nm)	0.007	0.006	0.005	0.006	0.001
Number of cells (cell/ml)	46222.95	41837.81	37452.68	41837.81	4385.135
% of control	38.27346	40.52885	31.01151	36.60461	4.973302
	BA-124$_1$	BA-124$_2$	BA-124$_3$	Mean	SD
OD (665 nm)	0.018	0.022	0.018	0.019333	0.002309
OD (750 nm)	0.01	0.014	0.01	0.011333	0.002309
Number of cells (cell/ml)	59378.35	76918.89	59378.35	65225.2	10127.04
% of control	49.16638	74.51236	49.16638	57.61504	14.63351
	BA-132$_1$	BA-132$_2$	BA-132$_3$	Mean	SD
OD (665 nm)	0.01	0.011	0.012	0.011	0.001
OD (750 nm)	0.003	0.004	0.005	0.004	0.001
Number of cells (cell/ml)	28682.41	33067.54	37452.68	33067.54	4385.135
% of control	23.74956	32.03297	31.01151	28.93135	4.516527
Navicula perminuta					
	Control$_1$	Control$_2$	Control$_3$	Mean	SD
OD (665 nm)	0.053	0.057	0.054	0.054667	0.002082
OD (750 nm)	0.045	0.048	0.046	0.046333	0.001528
Number of cells (cell/ml)	279724.2	298961.6	286136.7	288274.1	9795.178
	BA-120$_1$	BA-120$_2$	BA-120$_3$	Mean	SD
OD (665 nm)	0.068	0.068	0.069	0.068333	0.000577
OD (750 nm)	0.057	0.058	0.059	0.058	0.001
Number of cells (cell/ml)	356673.6	363086	369498.5	363086	6412.449
% of control	127.509	121.4491	129.1336	126.0306	4.049972
	BA-124$_1$	BA-124$_2$	BA-124$_3$	Mean	SD
OD (665 nm)	0.051	0.056	0.058	0.055	0.003606
OD (750 nm)	0.042	0.045	0.046	0.044333	0.002082
Number of cells (cell/ml)	260486.9	279724.2	286136.7	275449.2	13348.58

% of control	93.12274	93.56528	100	95.56267	3.849202
	BA-132$_1$	BA-132$_2$	BA-132$_3$	Mean	SD
OD (665 nm)	0.049	0.049	0.05	0.049333	0.000577
OD (750 nm)	0.04	0.04	0.041	0.040333	0.000577
Number of cells (cell/ml)	247662	247662	254074.4	249799.4	3702.229
% of control	88.53791	82.84074	88.79478	86.72448	3.365865
Nitzschia fonticola					
	Control$_1$	Control$_2$	Control$_3$	Mean	SD
OD (665 nm)	0.047	0.048	0.051	0.048667	0.002082
OD (750 nm)	0.037	0.038	0.04	0.038333	0.001528
Number of cells (cell/ml)	315703.3	323754.1	339855.7	326437.7	12297.79
	BA-120$_1$	BA-120$_2$	BA-120$_3$	Mean	SD
OD (665 nm)	0.027	0.031	0.033	0.030333	0.003055
OD (750 nm)	0.021	0.024	0.026	0.023667	0.002517
Number of cells (cell/ml)	186890.6	211043	227144.6	208359.4	20260.72
% of control	59.19819	65.1862	66.8356	63.74	4.018851
	BA-124$_1$	BA-124$_2$	BA-124$_3$	Mean	SD
OD (665 nm)	0.07	0.074	0.074	0.072667	0.002309
OD (750 nm)	0.05	0.054	0.055	0.053	0.002646
Number of cells (cell/ml)	420363.6	452566.8	460617.6	444516	21300.39
% of control	224.9249	214.4429	202.7861	214.0513	11.07462
	BA-132$_1$	BA-132$_2$	BA-132$_3$	Mean	SD
OD (665 nm)	0.029	0.029	0.028	0.028667	0.000577
OD (750 nm)	0.023	0.023	0.022	0.022667	0.000577
Number of cells (cell/ml)	202992.2	202992.2	194941.4	200308.6	4648.127
% of control	64.29841	62.69951	57.36006	61.45266	3.633339
Fistulifera saprophila					
	Control$_1$	Control$_2$	Control$_3$	Mean	SD
OD (665 nm)	0.075	0.078	0.078	0.077	0.001732
OD (750 nm)	0.062	0.065	0.064	0.063667	0.001528
Number of cells (cell/ml)	396483.8	418464.8	411137.8	408695.5	11192.15
	BA-120$_1$	BA-120$_2$	BA-120$_3$	Mean	SD
OD (665 nm)	0.059	0.058	0.053	0.056667	0.003215
OD (750 nm)	0.051	0.048	0.044	0.047667	0.003512
Number of cells (cell/ml)	315887	293906.1	264598.2	291463.8	25731.51
% of control	79.67211	70.23437	64.35754	71.42134	7.725975
	BA-124$_1$	BA-124$_2$	BA-124$_3$	Mean	SD
OD (665 nm)	0.057	0.056	0.058	0.057	0.001
OD (750 nm)	0.047	0.046	0.048	0.047	0.001
Number of cells (cell/ml)	286579.1	279252.1	293906.1	286579.1	7326.981
% of control	72.28015	66.73253	71.48603	70.16624	3.00007
	BA-132$_1$	BA-132$_2$	BA-132$_3$	Mean	SD
OD (665 nm)	0.034	0.036	0.035	0.035	0.001
OD (750 nm)	0.03	0.031	0.03	0.030333	0.000577
Number of cells (cell/ml)	162020.4	169347.4	162020.4	164462.8	4230.234
% of control	40.86432	40.46874	39.40782	40.24696	0.753154
Skeletonema marinoi					
	Control$_1$	Control$_2$	Control$_3$	Mean	SD
OD (665 nm)	0.14	0.155	0.144	0.146333	0.007767
OD (750 nm)	0.1	0.11	0.099	0.103	0.006083
Number of cells (cell/ml)	3885368	4266404	3847265	3999679	231774.9
	BA-120$_1$	BA-120$_2$	BA-120$_3$	Mean	SD

OD (665 nm)	0.113	0.13	0.12	0.121	0.008544
OD (750 nm)	0.076	0.09	0.07	0.078667	0.010263
Number of cells (cell/ml)	2970883	3504333	2742262	3072492	391064.5
% of control	76.46336	82.13786	71.27822	76.62648	5.431658
	BA-124$_1$	BA-124$_2$	BA-124$_3$	Mean	SD
OD (665 nm)	0.047	0.053	0.052	0.050667	0.003215
OD (750 nm)	0.033	0.037	0.035	0.035	0.002
Number of cells (cell/ml)	1332430	1484844	1408637	1408637	76207.1
% of control	34.29354	34.80319	36.614	35.23691	1.219515
	BA-132$_1$	BA-132$_2$	BA-132$_3$	Mean	SD
OD (665 nm)	0.026	0.028	0.022	0.025333	0.003055
OD (750 nm)	0.02	0.021	0.016	0.019	0.002646
Number of cells (cell/ml)	837084	875187.6	684669.8	798980.5	100812.5
% of control	21.54452	20.51347	17.79628	19.95142	1.936301

Table S2. Value of fluorescence of chlorophyll *a* (F_v/F_m parameter) of studied cyanobacteria and microalgae obtained after 7th day of the experiment for control and culture with the addition of filtrate obtained from cultures of cyanobacteria *Synechococcus* sp. (BA-120, BA-124 and BA-132).

***Planktolyngbya* sp.**					
	Control$_1$	Control$_2$	Control$_3$	Mean	SD
F_v/F_m	0.371	0.386	0.374	0.377	0.007937
	BA-120$_1$	BA-120$_2$	BA-120$_3$	Mean	SD
F_v/F_m	0.422	0.406	0.384	0.404	0.019079
% of control	113.7466	105.1813	102.6738	107.2006	5.806025
	BA-124$_1$	BA-124$_2$	BA-124$_3$	Mean	SD
F_v/F_m	0.465	0.464	0.445	0.458	0.011269
% of control	125.3369	120.2073	118.984	121.5094	3.370713
	BA-132$_1$	BA-132$_2$	BA-132$_3$	Mean	SD
F_v/F_m	0.456	0.424	0.399	0.426333	0.028572
% of control	122.9111	109.8446	106.6845	113.1467	8.602523
Aphanizomenon sp.					
	Control$_1$	Control$_2$	Control$_3$	Mean	SD
F_v/F_m	0.298	0.294	0.3	0.297333	0.003055
	BA-120$_1$	BA-120$_2$	BA-120$_3$	Mean	SD
F_v/F_m	0.338	0.333	0.334	0.335	0.002646
% of control	113.4228	113.2653	111.3333	112.6738	1.163563
	BA-124$_1$	BA-124$_2$	BA-124$_3$	Mean	SD
F_v/F_m	0.334	0.321	0.318	0.324333	0.008505
% of control	112.0805	109.1837	106	109.0881	3.041396
	BA-132$_1$	BA-132$_2$	BA-132$_3$	Mean	SD
F_v/F_m	0.356	0.344	0.327	0.342333	0.014572
% of control	119.4631	117.0068	109	115.1566	5.471417
***Nostoc* sp.**					
	Control$_1$	Control$_2$	Control$_3$	Mean	SD
F_v/F_m	0.502	0.497	0.498	0.499	0.002646
	BA-120$_1$	BA-120$_2$	BA-120$_3$	Mean	SD

F_v/F_m	0.208	0.222	0.22	0.216667	0.007572
% of control	41.43426	44.66801	44.17671	43.42633	1.742579
	BA-124$_1$	BA-124$_2$	BA-124$_3$	Mean	SD
F_v/F_m	0.115	0.188	0.097	0.133333	0.048191
% of control	22.90837	37.82696	19.47791	26.73775	9.755513
	BA-132$_1$	BA-132$_2$	BA-132$_3$	Mean	SD
F_v/F_m	0.143	0.1	0.19	0.144333	0.045015
% of control	28.48606	20.12072	38.15261	28.9198	9.023765
***Synechocystis* sp.**					
	Control$_1$	Control$_2$	Control$_3$	Mean	SD
F_v/F_m	0.449	0.417	0.43	0.432	0.016093
	BA-120$_1$	BA-120$_2$	BA-120$_3$	Mean	SD
F_v/F_m	0.393	0.346	0.36	0.366333	0.024132
% of control	87.52784	82.97362	83.72093	84.7408	2.442401
	BA-124$_1$	BA-124$_2$	BA-124$_3$	Mean	SD
F_v/F_m	0.524	0.412	0.438	0.458	0.058617
% of control	116.7038	98.80096	101.8605	105.7884	9.575977
	BA-132$_1$	BA-132$_2$	BA-132$_3$	Mean	SD
F_v/F_m	0.444	0.25	0.375	0.356333	0.098338
% of control	98.88641	59.95204	87.2093	82.01592	19.97999
***Phormidium* sp.**					
	Control$_1$	Control$_2$	Control$_3$	Mean	SD
F_v/F_m	0.439	0.439	0.435	0.437667	0.002309
	BA-120$_1$	BA-120$_2$	BA-120$_3$	Mean	SD
F_v/F_m	0.716	0.69	0.672	0.692667	0.022121
% of control	163.0979	157.1754	154.4828	158.252	4.407351
	BA-124$_1$	BA-124$_2$	BA-124$_3$	Mean	SD
F_v/F_m	0.692	0.632	0.672	0.665333	0.030551
% of control	157.631	143.9636	154.4828	152.0258	7.157321
	BA-132$_1$	BA-132$_2$	BA-132$_3$	Mean	SD
F_v/F_m	0.646	0.62	0.642	0.636	0.014
% of control	147.1526	141.2301	147.5862	145.323	3.551176
***Pseudanabaena* sp.**					
	Control$_1$	Control$_2$	Control$_3$	Mean	SD
F_v/F_m	0.441	0.418	0.424	0.427667	0.01193
	BA-120$_1$	BA-120$_2$	BA-120$_3$	Mean	SD
F_v/F_m	0.374	0.367	0.361	0.367333	0.006506
% of control	84.80726	87.79904	85.14151	85.91594	1.63936
	BA-124$_1$	BA-124$_2$	BA-124$_3$	Mean	SD
F_v/F_m	0.411	0.398	0.383	0.397333	0.014012
% of control	93.19728	95.21531	90.33019	92.91426	2.454828
	BA-132$_1$	BA-132$_2$	BA-132$_3$	Mean	SD
F_v/F_m	0.35	0.334	0.331	0.338333	0.010214
% of control	85.15815	83.9196	86.42298	85.16691	1.251712
Monoraphidium convolutum* var. *pseudosabulosum					
	Control$_1$	Control$_2$	Control$_3$	Mean	SD
F_v/F_m	0.772	0.771	0.772	0.771667	0.000577
	BA-120$_1$	BA-120$_2$	BA-120$_3$	Mean	SD
F_v/F_m	0.691	0.691	0.688	0.69	0.001732
% of control	89.50777	89.62387	89.11917	89.41694	0.264324

	BA-124$_1$	BA-124$_2$	BA-124$_3$	Mean	SD
***F*$_v$/*F*$_m$**	0.716	0.718	0.718	0.717333	0.001155
% of control	92.74611	93.12581	93.00518	92.95904	0.194009
	BA-132$_1$	BA-132$_2$	BA-132$_3$	Mean	SD
***F*$_v$/*F*$_m$**	0.645	0.642	0.637	0.641333	0.004041
% of control	83.54922	83.26848	82.51295	83.11022	0.535956
**Chl*orella fusca***					
	Control$_1$	Control$_2$	Control$_3$	Mean	SD
***F*$_v$/*F*$_m$**	0.255	0.248	0.249	0.250667	0.003786
	BA-120$_1$	BA-120$_2$	BA-120$_3$	Mean	SD
***F*$_v$/*F*$_m$**	0.195	0.193	0.19	0.192667	0.002517
% of control	76.47059	77.82258	76.30522	76.86613	0.832427
	BA-124$_1$	BA-124$_2$	BA-124$_3$	Mean	SD
***F*$_v$/*F*$_m$**	0.157	0.15	0.15	0.152333	0.004041
% of control	61.56863	60.48387	60.24096	60.76449	0.706917
	BA-132$_1$	BA-132$_2$	BA-132$_3$	Mean	SD
***F*$_v$/*F*$_m$**	0.118	0.106	0.108	0.110667	0.006429
% of control	46.27451	42.74194	43.37349	44.12998	1.883872
Kirchneriella obesa					
	Control$_1$	Control$_2$	Control$_3$	Mean	SD
***F*$_v$/*F*$_m$**	0.748	0.747	0.745	0.746667	0.001528
	BA-120$_1$	BA-120$_2$	BA-120$_3$	Mean	SD
***F*$_v$/*F*$_m$**	0.781	0.778	0.777	0.778667	0.002082
% of control	104.4118	104.1499	104.2953	104.2857	0.131181
	BA-124$_1$	BA-124$_2$	BA-124$_3$	Mean	SD
***F*$_v$/*F*$_m$**	0.735	0.74	0.739	0.738	0.002646
% of control	98.26203	99.06292	99.19463	98.83986	0.504729
	BA-132$_1$	BA-132$_2$	BA-132$_3$	Mean	SD
***F*$_v$/*F*$_m$**	0.724	0.724	0.723	0.723667	0.000577
% of control	96.79144	96.92102	97.04698	96.91981	0.127772
***Monoraphidium* sp.**					
	Control$_1$	Control$_2$	Control$_3$	Mean	SD
***F*$_v$/*F*$_m$**	0.584	0.578	0.583	0.581667	0.003215
	BA-120$_1$	BA-120$_2$	BA-120$_3$	Mean	SD
***F*$_v$/*F*$_m$**	0.347	0.339	0.343	0.343	0.004
% of control	59.41781	58.65052	58.83362	58.96732	0.400736
	BA-124$_1$	BA-124$_2$	BA-124$_3$	Mean	SD
***F*$_v$/*F*$_m$**	0.388	0.383	0.388	0.386333	0.002887
% of control	66.43836	66.26298	66.55232	66.41788	0.145752
	BA-132$_1$	BA-132$_2$	BA-132$_3$	Mean	SD
***F*$_v$/*F*$_m$**	0.24	0.244	0.237	0.240333	0.003512
% of control	41.09589	42.21453	40.6518	41.32074	0.805265
**Chl*orella* sp.**					
	Control$_1$	Control$_2$	Control$_3$	Mean	SD
***F*$_v$/*F*$_m$**	0.719	0.719	0.718	0.718667	0.000577
	BA-120$_1$	BA-120$_2$	BA-120$_3$	Mean	SD
***F*$_v$/*F*$_m$**	0.679	0.681	0.681	0.680333	0.001155
% of control	94.43672	94.71488	94.8468	94.66613	0.209341
	BA-124$_1$	BA-124$_2$	BA-124$_3$	Mean	SD
***F*$_v$/*F*$_m$**	0.629	0.623	0.623	0.625	0.003464
% of control	87.48261	86.64812	86.7688	86.96651	0.451012

	BA-132$_1$	BA-132$_2$	BA-132$_3$	Mean	SD
F_v/F_m	0.606	0.608	0.605	0.606333	0.001528
% of control	84.28373	84.56189	84.26184	84.36915	0.167275
Oocystis* cf. *submarina					
	Control$_1$	Control$_2$	Control$_3$	Mean	SD
F_v/F_m	0.688	0.671	0.672	0.677	0.009539
	BA-120$_1$	BA-120$_2$	BA-120$_3$	Mean	SD
F_v/F_m	0.87	0.848	0.854	0.857333	0.011372
% of control	126.4535	126.3785	127.0833	126.6385	0.387095
	BA-124$_1$	BA-124$_2$	BA-124$_3$	Mean	SD
F_v/F_m	0.82	0.817	0.811	0.816	0.004583
% of control	119.186	121.7586	120.6845	120.543	1.292084
	BA-132$_1$	BA-132$_2$	BA-132$_3$	Mean	SD
F_v/F_m	0.841	0.845	0.825	0.837	0.010583
% of control	122.2384	125.9314	122.7679	123.6459	1.996974
Cyclotella meneghiniana					
	Control$_1$	Control$_2$	Control$_3$	Mean	SD
F_v/F_m	0.828	0.826	0.824	0.826	0.002
	BA-120$_1$	BA-120$_2$	BA-120$_3$	Mean	SD
F_v/F_m	0.817	0.805	0.805	0.809	0.006928
% of control	98.6715	97.45763	97.69417	97.9411	0.643506
	BA-124$_1$	BA-124$_2$	BA-124$_3$	Mean	SD
F_v/F_m	0.814	0.81	0.811	0.811667	0.002082
% of control	98.30918	98.06295	98.42233	98.26482	0.183748
	BA-132$_1$	BA-132$_2$	BA-132$_3$	Mean	SD
F_v/F_m	0.815	0.809	0.807	0.810333	0.004163
% of control	98.42995	97.94189	97.93689	98.10291	0.283236
Amphora coffeaeformis					
	Control$_1$	Control$_2$	Control$_3$	Mean	SD
F_v/F_m	0.783	0.804	0.804	0.797	0.012124
	BA-120$_1$	BA-120$_2$	BA-120$_3$	Mean	SD
F_v/F_m	0.667	0.625	0.667	0.653	0.024249
% of control	85.18519	77.73632	82.9602	81.96057	3.823722
	BA-124$_1$	BA-124$_2$	BA-124$_3$	Mean	SD
F_v/F_m	0.556	0.556	0.5	0.537333	0.032332
% of control	71.00894	69.15423	62.18905	67.45074	4.650161
	BA-132$_1$	BA-132$_2$	BA-132$_3$	Mean	SD
F_v/F_m	0.4	0.571	0.571	0.514	0.098727
% of control	51.08557	71.0199	71.0199	64.37512	11.50909
Navicula perminuta					
	Control$_1$	Control$_2$	Control$_3$	Mean	SD
F_v/F_m	0.632	0.588	0.579	0.599667	0.028361
	BA-120$_1$	BA-120$_2$	BA-120$_3$	Mean	SD
F_v/F_m	0.613	0.608	0.614	0.611667	0.003215
% of control	96.99367	103.4014	106.0449	102.1466	4.654239
	BA-124$_1$	BA-124$_2$	BA-124$_3$	Mean	SD
F_v/F_m	0.493	0.487	0.489	0.489667	0.003055
% of control	78.00633	82.82313	84.45596	81.76181	3.353243
	BA-132$_1$	BA-132$_2$	BA-132$_3$	Mean	SD
F_v/F_m	0.582	0.595	0.605	0.594	0.011533

% of control	92.08861	101.1905	104.4905	99.25653	6.42315
Nitzschia fonticola					
	Control$_1$	Control$_2$	Control$_3$	Mean	SD
F_v/F_m	0.789	0.787	0.787	0.787667	0.001155
	BA-120$_1$	BA-120$_2$	BA-120$_3$	Mean	SD
F_v/F_m	0.714	0.74	0.729	0.727667	0.013051
% of control	90.4943	94.02795	92.63024	92.38416	1.779635
	BA-124$_1$	BA-124$_2$	BA-124$_3$	Mean	SD
F_v/F_m	0.427	0.434	0.431	0.430667	0.003512
% of control	54.11914	55.14612	54.76493	54.67673	0.519143
	BA-132$_1$	BA-132$_2$	BA-132$_3$	Mean	SD
F_v/F_m	0.4	0.438	0.429	0.422333	0.019858
% of control	50.69708	55.65438	54.5108	53.62076	2.595734
Fistulifera saprophila					
	Control$_1$	Control$_2$	Control$_3$	Mean	SD
F_v/F_m	0.795	0.802	0.798	0.798333	0.003512
	BA-120$_1$	BA-120$_2$	BA-120$_3$	Mean	SD
F_v/F_m	0.79	0.794	0.806	0.796667	0.008327
% of control	99.37107	99.00249	101.0025	99.79202	1.064384
	BA-124$_1$	BA-124$_2$	BA-124$_3$	Mean	SD
F_v/F_m	0.673	0.66	0.658	0.663667	0.008145
% of control	84.65409	82.29426	82.45614	83.13483	1.318202
	BA-132$_1$	BA-132$_2$	BA-132$_3$	Mean	SD
F_v/F_m	0.5	0.556	0.444	0.5	0.056
% of control	62.89308	69.32668	55.6391	62.61962	6.847889
Skeletonema marinoi					
	Control$_1$	Control$_2$	Control$_3$	Mean	SD
F_v/F_m	0.652	0.631	0.645	0.642667	0.010693
	BA-120$_1$	BA-120$_2$	BA-120$_3$	Mean	SD
F_v/F_m	0.664	0.646	0.654	0.654667	0.009018
% of control	101.8405	102.3772	101.3953	101.871	0.491626
	BA-124$_1$	BA-124$_2$	BA-124$_3$	Mean	SD
F_v/F_m	0.689	0.676	0.671	0.678667	0.009292
% of control	105.6748	107.1315	104.031	105.6125	1.551206
	BA-132$_1$	BA-132$_2$	BA-132$_3$	Mean	SD
F_v/F_m	0.333	0.333	0.333	0.333	0
% of control	48.33091	49.26036	49.62742	49.0729	0.668273

Table S3. Value of photosynthetic pigments (chlorophyll *a* and carotenoid pigments) of studied cyanobacteria and microalgae obtained after 7th day of the experiment for control and culture with the addition of filtrate obtained from cultures of cyanobacteria *Synechococcus* sp. (BA-120, BA-124 and BA-132).

***Planktolyngbya* sp.**					
	**Control$_1$**	**Control$_2$**	**Control$_3$**	**Mean**	**SD**
Chl *a* (ug/ml)	0.19474	0.243425	0.2186	0.218922	0.024344
Car (ug/ml)	0.192	0.226	0.178	0.198667	0.024685
Chl *a* (pg/cell)	0.018354	0.02262316	0.019501	0.02016	0.002209
Car (pg/cell)	0.018096	0.021003735	0.015879	0.018326	0.00257
	BA-120$_1$	BA-120$_2$	BA-120$_3$	Mean	SD

Chl *a* (ug/ml)	0.24746	0.32097	0.35483	0.307753	0.054892
Car (ug/ml)	0.12	0.13	0.122	0.124	0.005292
Chl *a* (pg/cell)	0.024719	0.032549	0.035181	0.030817	0.005442
Car (pg/cell)	0.011987	0.013183	0.012096	0.012422	0.000661
	$BA\text{-}124_1$	$BA\text{-}124_2$	$BA\text{-}124_3$	Mean	SD
Chl *a* (ug/ml)	0.39755	0.37869	0.3379	0.37138	0.030489
Car (ug/ml)	0.14	0.136	0.126	0.134	0.007211
Chl *a* (pg/cell)	0.042241	0.039606	0.034795	0.038881	0.003776
Car (pg/cell)	0.014875	0.014224	0.012975	0.014025	0.000966
	$BA\text{-}132_1$	$BA\text{-}132_2$	$BA\text{-}132_3$	Mean	SD
Chl *a* (ug/ml)	0.40062	0.31211	0.389655	0.367462	0.048248
Car (ug/ml)	0.136	0.11	0.128	0.124667	0.013317
Chl *a* (pg/cell)	0.044702	0.034537	0.041734	0.040324	0.005227
Car (pg/cell)	0.015175	0.012172	0.01371	0.013686	0.001502
Aphanizomenon sp.					
	$Control_1$	$Control_2$	$Control_3$	Mean	SD
Chl *a* (ug/ml)	0.195705	0.255355	0.303075	0.251378	0.053795
Car (ug/ml)	0.11	0.13	0.11	0.116667	0.011547
Chl *a* (pg/cell)	0.559251	0.689979384	0.804324	0.684518	0.122627
Car (pg/cell)	0.314339	0.351265179	0.291926	0.319177	0.029964
	$BA\text{-}120_1$	$BA\text{-}120_2$	$BA\text{-}120_3$	Mean	SD
Chl *a* (ug/ml)	0.27825	0.267285	0.348865	0.298133	0.044276
Car (ug/ml)	0.148	0.158	0.148	0.151333	0.005774
Chl *a* (pg/cell)	0.795134	0.684924	0.878849	0.786302	0.097263
Car (pg/cell)	0.422928	0.404879	0.372836	0.400214	0.02537
	$BA\text{-}124_1$	$BA\text{-}124_2$	$BA\text{-}124_3$	Mean	SD
Chl *a* (ug/ml)	0.43141	0.4522	0.52378	0.46913	0.048456
Car (ug/ml)	0.124	0.138	0.142	0.134667	0.009452
Chl *a* (pg/cell)	0.971704	0.921	1.112441	1.001715	0.099186
Car (pg/cell)	0.279297	0.281066	0.30159	0.287317	0.012392
	$BA\text{-}132_1$	$BA\text{-}132_2$	$BA\text{-}132_3$	Mean	SD
Chl *a* (ug/ml)	0.434305	0.50492	0.52185	0.487025	0.046435
Car (ug/ml)	0.124	0.152	0.15	0.142	0.01562
Chl *a* (pg/cell)	1.024732	1.028375	1.108341	1.053816	0.047255
Car (pg/cell)	0.292575	0.30958	0.31858	0.306912	0.013206
***Nostoc* sp.**					
	$Control_1$	$Control_2$	$Control_3$	Mean	SD
Chl *a* (ug/ml)	0.8001	1.337915	0.83203	0.990015	0.301713
Car (ug/ml)	0.242	0.268	0.2	0.236667	0.034312
Chl *a* (pg/cell)	-0.43701	-0.69469	-0.41279	-0.51483	0.156233
Car (pg/cell)	0.118188	0.20363195	0.125872	0.149231	0.047269
	$BA\text{-}120_1$	$BA\text{-}120_2$	$BA\text{-}120_3$	Mean	SD
Chl *a* (ug/ml)	0.139125	0.15009	0.144125	0.144447	0.00549
Car (ug/ml)	0.048	0.054	0.046	0.049333	0.004163
Chl *a* (pg/cell)	-0.08361	-0.07893	-0.07618	-0.07957	0.003757
Car (pg/cell)	0.067455	0.072772	0.071258	0.070495	0.002739
	$BA\text{-}124_1$	$BA\text{-}124_2$	$BA\text{-}124_3$	Mean	SD
Chl *a* (ug/ml)	0.08737	0.1093	0.1093	0.10199	0.012661
Car (ug/ml)	0.03	0.04	0.04	0.036667	0.005774
Chl *a* (pg/cell)	-0.07922	-0.06986	-0.06986	-0.07298	0.005404
Car (pg/cell)	0.040038	0.046675	0.046675	0.044463	0.003832
	$BA\text{-}132_1$	$BA\text{-}132_2$	$BA\text{-}132_3$	Mean	SD

Chl *a* (ug/ml)	0.09044	0.05658	0.02272	0.05658	0.03386
Car (ug/ml)	0.032	0.028	0.014	0.024667	0.009452
Chl *a* (pg/cell)	-0.05143	-0.05529	-0.05915	-0.05529	0.00386
Car (pg/cell)	0.03929	0.026912	0.010225	0.025476	0.014586
***Synechocystis* sp.**					
	Control$_1$	Control$_2$	Control$_3$	Mean	SD
Chl *a* (ug/ml)	0.186845	0.2286	0.210705	0.208717	0.020948
Car (ug/ml)	0.092	0.122	0.082	0.098667	0.020817
Chl *a* (pg/cell)	0.014521	0.01732481	0.015035	0.015627	0.001493
Car (pg/cell)	0.00715	0.009245961	0.005851	0.007416	0.001713
	BA-120$_1$	BA-120$_2$	BA-120$_3$	Mean	SD
Chl *a* (ug/ml)	0.031755	0.04465	0.074475	0.050293	0.021912
Car (ug/ml)	0.026	0.038	0.032	0.032	0.006
Chl *a* (pg/cell)	0.004668	0.007076	0.011219	0.007654	0.003313
Car (pg/cell)	0.003822	0.006022	0.004821	0.004888	0.001101
	BA-124$_1$	BA-124$_2$	BA-124$_3$	Mean	SD
Chl *a* (ug/ml)	0.038685	0.050615	0.07158	0.053627	0.016653
Car (ug/ml)	0.028	0.028	0.04	0.032	0.006928
Chl *a* (pg/cell)	0.006294	0.008699	0.011965	0.008986	0.002846
Car (pg/cell)	0.004555	0.004812	0.006686	0.005351	0.001163
	BA-132$_1$	BA-132$_2$	BA-132$_3$	Mean	SD
Chl *a* (ug/ml)	0.026755	0.03272	0.050615	0.036697	0.012417
Car (ug/ml)	0.028	0.028	0.03	0.028667	0.001155
Chl *a* (pg/cell)	0.005728	0.007533	0.011229	0.008163	0.002805
Car (pg/cell)	0.005994	0.006447	0.006656	0.006365	0.000338
***Phormidium* sp.**					
	Control$_1$	Control$_2$	Control$_3$	Mean	SD
Chl *a* (ug/ml)	1.05177	1.12721	1.059665	1.079548	0.041465
Car (ug/ml)	0.242	0.264	0.244	0.25	0.012166
Chl *a* (pg/cell)	0.025945	0.026573032	0.028843	0.02712	0.001524
Car (pg/cell)	0.230088	0.234206581	0.230261	0.231519	0.002329
	BA-120$_1$	BA-120$_2$	BA-120$_3$	Mean	SD
Chl *a* (ug/ml)	0.181845	0.173863	0.16588	0.173863	0.007983
Car (ug/ml)	0.064	0.066	0.068	0.066	0.002
Chl *a* (pg/cell)	0.011803	0.01079	0.013873	0.012155	0.001571
Car (pg/cell)	0.351948	0.37961	0.409935	0.380498	0.029004
	BA-124$_1$	BA-124$_2$	BA-124$_3$	Mean	SD
Chl *a* (ug/ml)	0.146055	0.12316	0.134608	0.134608	0.011448
Car (ug/ml)	0.064	0.046	0.055	0.055	0.009
Chl *a* (pg/cell)	0.013292	0.009911	0.01185	0.011684	0.001697
Car (pg/cell)	0.438191	0.373498	0.408595	0.406761	0.032386
	BA-132$_1$	BA-132$_2$	BA-132$_3$	Mean	SD
Chl *a* (ug/ml))	0.15202	0.19281	0.17588	0.17357	0.020493
Car (ug/ml)	0.06	0.08	0.074	0.071333	0.010263
Chl *a* (pg/cell)	0.011822	0.01863	0.019169	0.01654	0.004095
Car (pg/cell)	0.394685	0.414916	0.420741	0.410114	0.013676
***Pseudanabaena* sp.**					
	Control$_1$	Control$_2$	Control$_3$	Mean	SD
Chl *a* (ug/ml)	0.56764	0.62229	0.594965	0.594965	0.027325
Car (ug/ml)	0.252	0.254	0.253	0.253	0.001
Chl *a* (pg/cell)	0.027752	0.029336156	0.028048	0.028379	0.000842

Car (pg/cell)	0.01232	0.011974134	0.011927	0.012074	0.000215
	BA-120$_1$	BA-120$_2$	BA-120$_3$	Mean	SD
Chl *a* (ug/ml)	0.49185	0.44334	0.343865	0.426352	0.075441
Car (ug/ml)	0.146	0.198	0.148	0.164	0.029462
Chl *a* (pg/cell)	0.027057	0.025267	0.024115	0.025479	0.001483
Car (pg/cell)	0.008032	0.011284	0.010379	0.009898	0.001679
	BA-124$_1$	BA-124$_2$	BA-124$_3$	Mean	SD
Chl *a* (ug/ml)	0.52685	0.44141	0.341935	0.436732	0.092546
Car (ug/ml)	0.208	0.214	0.192	0.204667	0.011372
Chl *a* (pg/cell)	0.023842	0.020325	0.01593	0.020032	0.003964
Car (pg/cell)	0.009413	0.009854	0.008945	0.009404	0.000454
	BA-132$_1$	BA-132$_2$	BA-132$_3$	Mean	SD
Chl *a* (ug/ml)	0.34097	0.3579	0.353865	0.350912	0.008843
Car (ug/ml)	0.12	0.124	0.13	0.124667	0.005033
Chl *a* (pg/cell)	0.020621	0.022505	0.020766	0.021297	0.001049
Car (pg/cell)	0.007257	0.007797	0.007629	0.007561	0.000276
Monoraphidium convolutum* var. *pseudosabulosum					
	Control$_1$	Control$_2$	Control$_3$	Mean	SD
Chl *a* (ug/ml)	2.30897	1.646325	2.45055	2.135282	0.429325
Car (ug/ml)	0.704	0.6	0.752	0.685333	0.0777
Chl *a* (pg/cell)	0.285330823	0.182113	0.265939	0.244461	0.054858
Car (pg/cell)	0.086996756	0.066371	0.081609	0.078325	0.010698
	BA-120$_1$	BA-120$_2$	BA-120$_3$	Mean	SD
Chl *a* (ug/ml)	0.96115	0.78185	0.904395	0.882465	0.09164
Car (ug/ml)	0.368	0.31	0.362	0.346667	0.031896
Chl *a* (pg/cell)	0.132224	0.102293	0.115315	0.11661	0.015008
Car (pg/cell)	0.050625	0.040559	0.046157	0.04578	0.005044
	BA-124$_1$	BA-124$_2$	BA-124$_3$	Mean	SD
Chl *a* (ug/ml)	0.735095	0.6972	0.261845	0.564713	0.262975
Car (ug/ml)	0.298	0.28	0.134	0.237333	0.089941
Chl *a* (pg/cell)	0.094631	0.090333	0.031674	0.072213	0.035173
Car (pg/cell)	0.038363	0.036278	0.016209	0.030283	0.012233
	BA-132$_1$	BA-132$_2$	BA-132$_3$	Mean	SD
Chl *a* (ug/ml)	0.117545	0.10851	0.102545	0.109533	0.007552
Car (ug/ml)	0.08	0.078	0.07	0.076	0.005292
Chl *a* (pg/cell)	0.020188	0.018478	0.016889	0.018519	0.00165
Car (pg/cell)	0.01374	0.013283	0.011529	0.012851	0.001167
**Chl*orella fusca***					
	Control$_1$	Control$_2$	Control$_3$	Mean	SD
Chl *a* (ug/ml)	0.097545	0.202985	0.124475	0.141668	0.054782
Car (ug/ml)	0.078	0.104	0.098	0.093333	0.013614
Chl *a* (pg/cell)	0.029856	0.055963942	0.036491	0.04077	0.01357
Car (pg/cell)	0.023874	0.028673301	0.028729	0.027092	0.002787
	BA-120$_1$	BA-120$_2$	BA-120$_3$	Mean	SD
Chl *a* (ug/ml)	0.114475	0.097545	0.068685	0.093568	0.023153
Car (ug/ml)	0.102	0.088	0.07	0.086667	0.016042
Chl *a* (pg/cell)	0.034135	0.030667	0.020748	0.028517	0.006948
Car (pg/cell)	0.030416	0.027666	0.021146	0.026409	0.004761
	BA-124$_1$	BA-124$_2$	BA-124$_3$	Mean	SD
Chl *a* (ug/ml)	-0.005	0.05772	0.04772	0.03348	0.033698
Car (ug/ml)	0.054	0.064	0.066	0.061333	0.006429

Chl *a* (pg/cell)	0.016609	0.01874	0.014478	0.016609	0.002131
Car (pg/cell)	0.01737	0.020779	0.020024	0.019391	0.001791
	BA-132 1	BA-132 2	BA-132 3	Mean	SD
Chl *a* (ug/ml)	0.04772	0.046755	0.058685	0.051053	0.006627
Car (ug/ml)	0.062	0.068	0.08	0.07	0.009165
Chl *a* (pg/cell)	0.01423	0.013535	0.018704	0.01549	0.002805
Car (pg/cell)	0.018488	0.019686	0.025497	0.021223	0.003749
Kirchneriella obesa					
	Control 1	Control 2	Control 3	Mean	SD
Chl *a* (ug/ml)	1.40405	1.64914	1.53361	1.528933	0.122612
Car (ug/ml)	0.446	0.552	0.564	0.520667	0.064941
Chl *a* (pg/cell)	0.295612994	0.322839	0.287687	0.302047	0.018438
Car (pg/cell)	0.093902208	0.108061	0.1058	0.102588	0.007606
	BA-120 1	BA-120 2	BA-120 3	Mean	SD
Chl *a* (ug/ml)	0.542725	0.622375	0.554655	0.573252	0.042958
Car (ug/ml)	0.268	0.294	0.244	0.268667	0.025007
Chl *a* (pg/cell)	0.136687	0.164429	0.144647	0.148588	0.014284
Car (pg/cell)	0.067497	0.077673	0.063632	0.069601	0.007253
	BA-124 1	BA-124 2	BA-124 3	Mean	SD
Chl *a* (ug/ml)	0.3286	0.29974	0.278775	0.302372	0.025017
Car (ug/ml)	0.206	0.212	0.198	0.205333	0.007024
Chl *a* (pg/cell)	0.106681	0.097311	0.085689	0.09656	0.010516
Car (pg/cell)	0.066878	0.068826	0.060861	0.065522	0.004153
	BA-132 1	BA-132 2	BA-132 3	Mean	SD
Chl *a* (ug/ml)	0.16544	0.227195	0.119475	0.170703	0.054053
Car (ug/ml)	0.086	0.126	0.088	0.1	0.022539
Chl *a* (pg/cell)	0.068931	0.089149	0.038326	0.065469	0.025588
Car (pg/cell)	0.035832	0.049441	0.028229	0.037834	0.010747
***Monoraphidium* sp.**					
	Control 1	Control 2	Control 3	Mean	SD
Chl *a* (ug/ml)	1.568875	1.850895	1.673525	1.697765	0.142564
Car (ug/ml)	0.496	0.634	0.55	0.56	0.069541
Chl *a* (pg/cell)	0.361472	0.376386055	0.36464	0.367499	0.007858
Car (pg/cell)	0.114279	0.128926146	0.119838	0.121014	0.007394
	BA-120 1	BA-120 2	BA-120 3	Mean	SD
Chl *a* (ug/ml)	0.459215	0.47018	0.315705	0.415033	0.086195
Car (ug/ml)	0.296	0.312	0.254	0.287333	0.029956
Chl *a* (pg/cell)	0.118724	0.119532	0.076194	0.104817	0.024791
Car (pg/cell)	0.076527	0.079318	0.061302	0.072382	0.009697
	BA-124 1	BA-124 2	BA-124 3	Mean	SD
Chl *a* (ug/ml)	0.570795	0.48711	0.55983	0.539245	0.045482
Car (ug/ml)	0.336	0.312	0.348	0.332	0.01833
Chl *a* (pg/cell)	0.144628	0.135595	0.143279	0.141168	0.004873
Car (pg/cell)	0.085136	0.086851	0.089065	0.087017	0.00197
	BA-132 1	BA-132 2	BA-132 3	Mean	SD
Chl *a* (ug/ml)	0.05772	0.073685	0.073685	0.068363	0.009217
Car (ug/ml)	0.084	0.08	0.102	0.088667	0.011719
Chl *a* (pg/cell)	0.017541	0.02137	0.022215	0.020375	0.002491
Car (pg/cell)	0.025527	0.023202	0.030752	0.026494	0.003867
**Chl*orella* sp.**					
	Control 1	Control 2	Control 3	Mean	SD
Chl *a* (ug/ml)	0.06693	0.044738	0.010965	0.040878	0.028181

Car (ug/ml)	0.056	0.032	0.036	0.041333	0.012858
Chl *a* (pg/cell)	0.039255079	0.039255	0.039255	0.039255	0
Car (pg/cell)	0.03284453	0.016554	0.01936	0.02292	0.008709
	BA-120$_1$	BA-120$_2$	BA-120$_3$	Mean	SD
Chl *a* (ug/ml)	0.020965	0.026843	0.025	0.024269	0.003006
Car (ug/ml)	0.018	0.026	0.026	0.023333	0.004619
Chl *a* (pg/cell)	0.015679	0.016913	0.018147	0.016913	0.001234
Car (pg/cell)	0.013461	0.018674	0.018873	0.017003	0.003068
	BA-124$_1$	BA-124$_2$	BA-124$_3$	Mean	SD
Chl *a* (ug/ml)	0.020965	0.02386	0.020965	0.02193	0.001671
Car (ug/ml)	0.016	0.008	0.024	0.016	0.008
Chl *a* (pg/cell)	0.017455	0.017121	0.016787	0.017121	0.000334
Car (pg/cell)	0.013321	0.006368	0.019217	0.012969	0.006432
	BA-132$_1$	BA-132$_2$	BA-132$_3$	Mean	SD
Chl *a* (ug/ml)	0.015965	0.008948	0	0.008304	0.008002
Car (ug/ml)	0.016	0.014	0.012	0.014	0.002
Chl *a* (pg/cell)	0.013499	0.013499	0.013499	0.013499	0
Car (pg/cell)	0.013529	0.011934	0.010339	0.011934	0.001595
Oocystis* cf. *submarina					
	Control$_1$	Control$_2$	Control$_3$	Mean	SD
Chl *a* (ug/ml)	0.20623	0.185265	0.187195	0.192897	0.011587
Car (ug/ml)	0.21	0.18	0.178	0.189333	0.017926
Chl *a* (pg/cell)	0.206347	0.18537	0.177731	0.189816	0.014817
Car (pg/cell)	0.210119	0.180102	0.169001	0.186408	0.021272
	BA-120$_1$	BA-120$_2$	BA-120$_3$	Mean	SD
Chl *a* (ug/ml)	0.04079	0.06965	0.039825	0.050088	0.016948
Car (ug/ml)	0.058	0.09	0.078	0.075333	0.016166
Chl *a* (pg/cell)	0.05534	0.090767	0.054529	0.066879	0.020692
Car (pg/cell)	0.078689	0.117287	0.106798	0.100925	0.019958
	BA-124$_1$	BA-124$_2$	BA-124$_3$	Mean	SD
Chl *a* (ug/ml)	0.046755	0.039825	0.034825	0.040468	0.005991
Car (ug/ml)	0.066	0.068	0.05	0.061333	0.009866
Chl *a* (pg/cell)	0.071235	0.057439	0.049985	0.059553	0.010782
Car (pg/cell)	0.100556	0.098075	0.071766	0.090132	0.015954
	BA-132$_1$	BA-132$_2$	BA-132$_3$	Mean	SD
Chl *a* (ug/ml)	0.068685	0.056755	0.073685	0.066375	0.008698
Car (ug/ml)	0.07	0.074	0.072	0.072	0.002
Chl *a* (pg/cell)	0.105186	0.091141	0.115826	0.104051	0.012382
Car (pg/cell)	0.1072	0.118835	0.113178	0.113071	0.005818
Cyclotella meneghiniana					
	Control$_1$	Control$_2$	Control$_3$	Mean	SD
Chl *a* (ug/ml)	0.875625	0.954135	1.062645	0.964135	0.09391
Car (ug/ml)	0.216	0.23	0.272	0.239333	0.029143
Chl *a* (pg/cell)	1.613067	1.534442398	1.733412	1.626974	0.100211
Car (pg/cell)	0.397913	0.3698866	0.443693	0.403831	0.037257
	BA-120$_1$	BA-120$_2$	BA-120$_3$	Mean	SD
Chl *a* (ug/ml)	0.7015	0.7065	0.74922	0.719073	0.026227
Car (ug/ml)	0.186	0.21	0.216	0.204	0.015875
Chl *a* (pg/cell)	1.541497	1.44131	1.402884	1.461897	0.071563
Car (pg/cell)	0.408722	0.428415	0.404451	0.413863	0.012782
	BA-124$_1$	BA-124$_2$	BA-124$_3$	Mean	SD
Chl *a* (ug/ml)	0.47369	0.556235	0.59106	0.540328	0.06028

Car (ug/ml)	0.138	0.16	0.174	0.157333	0.018148
Chl *a* (pg/cell)	1.040901	1.246321	1.298813	1.195345	0.136303
Car (pg/cell)	0.303245	0.358502	0.382353	0.348033	0.040579
	BA-132$_1$	BA-132$_2$	BA-132$_3$	Mean	SD
Chl *a* (ug/ml)	0.448865	0.4179	0.39097	0.419245	0.028971
Car (ug/ml)	0.134	0.122	0.11	0.122	0.012
Chl *a* (pg/cell)	0.932409	0.900933	0.827226	0.886856	0.053986
Car (pg/cell)	0.278353	0.263015	0.232741	0.258036	0.02321
Amphora coffeaeformis					
	Control$_1$	Control$_2$	Control$_3$	Mean	SD
Chl *a* (ug/ml)	0.54641	0.430795	0.52676	0.501322	0.061863
Car (ug/ml)	0.176	0.13	0.166	0.157333	0.024194
Chl *a* (pg/cell)	4.524376	4.173169	4.361671	4.353072	0.175761
Car (pg/cell)	1.457313	1.259327	1.374511	1.363717	0.099433
	BA-120$_1$	BA-120$_2$	BA-120$_3$	Mean	SD
Chl *a* (ug/ml)	0.003945	-0.01246	0.014735	0.002073	0.013694
Car (ug/ml)	0.014	0.018	0.02	0.017333	0.003055
Chl *a* (pg/cell)	0.085347	0.239389	0.39343	0.239389	0.154041
Car (pg/cell)	0.30288	0.430233	0.534007	0.422373	0.115764
	BA-124$_1$	BA-124$_2$	BA-124$_3$	Mean	SD
Chl *a* (ug/ml)	0.046755	0.029735	0.07351	0.05	0.022067
Car (ug/ml)	0.02	0.02	0.026	0.022	0.003464
Chl *a* (pg/cell)	0.812285	0.386576	1.237993	0.812285	0.425709
Car (pg/cell)	0.336823	0.260014	0.43787	0.344902	0.089203
	BA-132$_1$	BA-132$_2$	BA-132$_3$	Mean	SD
Chl *a* (ug/ml)	0.04772	0.0107	0.041755	0.033392	0.019877
Car (ug/ml)	0.006	0.014	0.008	0.009333	0.004163
Chl *a* (pg/cell)	1.663738	0.32358	1.114874	1.034064	0.673723
Car (pg/cell)	0.209187	0.423376	0.213603	0.282055	0.122407
Navicula perminuta					
	Control$_1$	Control$_2$	Control$_3$	Mean	SD
Chl *a* (ug/ml)	0.357285	0.253775	0.30553	0.30553	0.051755
Car (ug/ml)	0.136	0.098	0.116	0.116667	0.019009
Chl *a* (pg/cell)	1.277276	0.848855	1.067777	1.064636	0.214228
Car (pg/cell)	0.486193	0.327801	0.405401	0.406465	0.079201
	BA-120$_1$	BA-120$_2$	BA-120$_3$	Mean	SD
Chl *a* (ug/ml)	0.438865	0.39211	0.460795	0.43059	0.035082
Car (ug/ml)	0.16	0.148	0.156	0.154667	0.00611
Chl *a* (pg/cell)	1.230439	1.079937	1.247082	1.185819	0.092074
Car (pg/cell)	0.448589	0.407617	0.422194	0.426133	0.020768
	BA-124$_1$	BA-124$_2$	BA-124$_3$	Mean	SD
Chl *a* (ug/ml)	0.196055	0.18509	0.201055	0.194067	0.008166
Car (ug/ml)	0.074	0.07	0.074	0.072667	0.002309
Chl *a* (pg/cell)	0.752648	0.661687	0.702654	0.705663	0.045555
Car (pg/cell)	0.284083	0.250246	0.258618	0.264316	0.017623
	BA-132$_1$	BA-132$_2$	BA-132$_3$	Mean	SD
Chl *a* (ug/ml)	0.1393	0.12237	0.106405	0.122692	0.01645
Car (ug/ml)	0.06	0.048	0.042	0.05	0.009165
Chl *a* (pg/cell)	0.56246	0.494101	0.418795	0.491785	0.071861
Car (pg/cell)	0.242266	0.193813	0.165306	0.200461	0.038908
Nitzschia fonticola					

	Control1	Control2	Control3	Mean	SD
Chl *a* (ug/ml)	0.253775	0.236845	0.277635	0.256085	0.020493
Car (ug/ml)	0.078	0.07	0.084	0.077333	0.007024
Chl *a* (pg/cell)	0.80384	0.731558	0.81692	0.784106	0.045975
Car (pg/cell)	0.247067	0.216213	0.247164	0.236815	0.017841
	BA-1201	BA-1202	BA-1203	Mean	SD
Chl *a* (ug/ml)	0.05772	0.08658	0.075615	0.073305	0.014568
Car (ug/ml)	0.026	0.03	0.026	0.027333	0.002309
Chl *a* (pg/cell)	0.308844	0.410248	0.332894	0.350662	0.052986
Car (pg/cell)	0.139119	0.142151	0.114465	0.131911	0.015185
	BA-1241	BA-1242	BA-1243	Mean	SD
Chl *a* (ug/ml)	0.097545	0.06965	0.133335	0.100177	0.031924
Car (ug/ml)	0.046	0.032	0.058	0.045333	0.013013
Chl *a* (pg/cell)	0.232049	0.1539	0.28947	0.22514	0.068049
Car (pg/cell)	0.109429	0.070708	0.125918	0.102018	0.028341
	BA-1321	BA-1322	BA-1323	Mean	SD
Chl *a* (ug/ml)	0.022895	0.022895	0.01693	0.020907	0.003444
Car (ug/ml)	0.014	0.014	0.014	0.014	1.73E-18
Chl *a* (pg/cell)	0.112788	0.112788	0.086847	0.104141	0.014977
Car (pg/cell)	0.068968	0.068968	0.071816	0.069918	0.001644
Fistulifera saprophila					
	Control1	Control2	Control3	Mean	SD
Chl *a* (ug/ml)	0.75922	0.800975	0.79001	0.783402	0.021648
Car (ug/ml)	0.238	0.256	0.256	0.25	0.010392
Chl *a* (pg/cell)	1.914883	1.91408	1.921521	1.916828	0.004084
Car (pg/cell)	0.600277	0.61176	0.622662	0.611566	0.011194
	BA-1201	BA-1202	BA-1203	Mean	SD
Chl *a* (ug/ml)	0.267635	0.24974	0.23281	0.250062	0.017415
Car (ug/ml)	0.104	0.104	0.096	0.101333	0.004619
Chl *a* (pg/cell)	0.847249	0.849727	0.879862	0.858946	0.018156
Car (pg/cell)	0.329232	0.353855	0.362814	0.348633	0.017389
	BA-1241	BA-1242	BA-1243	Mean	SD
Chl *a* (ug/ml)	0.313425	0.301495	0.260705	0.291875	0.027645
Car (ug/ml)	0.11	0.11	0.098	0.106	0.006928
Chl *a* (pg/cell)	1.093677	1.079652	0.887035	1.020121	0.115469
Car (pg/cell)	0.383838	0.393909	0.33344	0.370396	0.032398
	BA-1321	BA-1322	BA-1323	Mean	SD
Chl *a* (ug/ml)	0.03579	0.034825	0.034825	0.035147	0.000557
Car (ug/ml)	0.02	0.018	0.02	0.019333	0.001155
Chl *a* (pg/cell)	0.220898	0.205642	0.214942	0.213827	0.007689
Car (pg/cell)	0.123441	0.10629	0.123441	0.117724	0.009902
Skeletonema marinoi					
	Control1	Control2	Control3	Mean	SD
Chl *a* (ug/ml)	1.368525	1.16361	1.28598	1.272705	0.1031
Car (ug/ml)	0.454	0.426	0.468	0.449333	0.021385
Chl *a* (pg/cell)	0.352225	0.272738	0.334258	0.31974	0.041685
Car (pg/cell)	0.116849	0.09985	0.121645	0.112781	0.011453
	BA-1201	BA-1202	BA-1203	Mean	SD
Chl *a* (ug/ml)	0.718605	1.05203	1.120715	0.963783	0.21509
Car (ug/ml)	0.256	0.368	0.402	0.342	0.076394
Chl *a* (pg/cell)	0.241883	0.300208	0.408683	0.316925	0.084647
Car (pg/cell)	0.08617	0.105013	0.146594	0.112592	0.030917

	BA-124$_1$	BA-124$_2$	BA-124$_3$	Mean	SD
Chl *a* (ug/ml)	0.36325	0.32246	0.35132	0.345677	0.020972
Car (ug/ml)	0.11	0.112	0.13	0.117333	0.011015
Chl *a* (pg/cell)	0.272622	0.217168	0.249404	0.246398	0.027849
Car (pg/cell)	0.082556	0.075429	0.092288	0.083424	0.008463
	BA-132$_1$	BA-132$_2$	BA-132$_3$	Mean	SD
Chl *a* (ug/ml)	0.05772	0.05272	0.02886	0.046433	0.015423
Car (ug/ml)	0.024	0.02	0.012	0.018667	0.00611
Chl *a* (pg/cell)	0.068954	0.060239	0.042152	0.057115	0.013671
Car (pg/cell)	0.028671	0.022852	0.017527	0.023017	0.005574

Table S4. Phytochemicals identified in different phenotypes of picocyanobacteria from the genus *Synechococcus* Type 1 (green strain), Type 2 (red strain), and Type 3a (brown strain) by GC-MS. The compounds found in both the f/2 medium and picocyanobacterial cultures are highlighted in italics.

**Name of compound**	**RT**	**Molecular**	**MW**	**Type 1**		**Type 2**		**Type 3a**		**f/2 medium**	
				**Peak Area %**	**SI**	**Peak Area %**	**SI**	**Peak Area %**	**SI**	**Peak Area %**	**SI**
Silanediol, dimethyl-	4.301	$C_2H_8O_2Si$	92	ND	ND	ND	ND	13.03	97	ND	ND
Oxime-, methoxy-phenyl-	8.444	$C_8H_9NO_2$	151	0.79	83	12.91	83	4.51	83	ND	ND
Cyclotetrasiloxane, octamethyl-	9.554	$C_8H_{24}O_4Si_4$	296	ND	ND	3.52	91	ND	ND	ND	ND
trisiloxane, 1,1,1,5,5,5-hexamethyl-3-[(trimethylsilyl)oxy]-	9.563	$C_9H_{28}O_3Si_4$	296	ND	ND	ND	ND	3.53	93	ND	ND
Decane, 3,7-dimethyl-	10.214	$C_{12}H_{26}$	170	ND	ND	ND	ND	ND	ND	2.53	93
Cyclotrisiloxane, hexamethyl-	10.325	$C_6H_{18}O_3Si_3$	222	ND	ND	5.52	92	ND	ND	ND	ND
Nonanal	10.654	$C_9H_{18}O$	142	ND	ND	ND	ND	ND	ND	3.96	89
Cyclopentasiloxane, decamethyl-	11.079	$C_{10}H_{30}O_5Si_5$	370	1.03	84	7.39	83	6.75	83	8.16	83
Acetoxyacetic acid, 5-tetradecyl ester	11.323	$C_{18}H_{34}O_4$	314	ND	ND	2.74	79	ND	ND	ND	ND
Decanal	11.565	$C_{10}H_{20}O$	156	ND	ND	ND	ND	ND	ND	1.88	93
Bicyclo[3.1.1]hept-3-en-2-one, 4,6,6-trimethyl-, (1S)-	11.692	$C_{10}H_{14}O$	150	ND	ND	4.65	94	3.99	96	ND	ND
Artedouglasia oxide A	11.800	$C_{15}H_{22}O_3$	250	ND	ND	3.66	78	ND	ND	ND	ND
Nonane, 5-(2-methylpropyl)-	12.185	$C_{13}H_{28}$	184	ND	ND	ND	ND	ND	ND	2.72	93
3-tert-Butyl-2-pyrazolin-5-one	12.271	$C_7H_{12}N_2O$	140	0.77	86	ND	ND	ND	ND	ND	ND
Cyclohexasiloxane, dodecamethyl-	12.489	$C_{12}H_{36}O_6Si_6$	444	1.89	90	14.67	90	12.39	90	8.06	90
Nonane, 5-(2-methylpropyl)-	12.536	$C_{13}H_{28}$	184	ND	ND	ND	ND	ND	ND	2.05	91
2,2,6,7-Tetramethyl-10-oxatricyclo[4.3.0.1(1,7)]decan-5-one	12.748	$C_{13}H_{20}O_2$	208	0.80	79	ND	ND	ND	ND	ND	ND
Naphthalene, decahydro-1,4a-dimethyl-7-(1-methylethyl)-,[1S-(1.alpha.,4a.alpha.,7.alpha., 8a.beta.)]-	12.750	$C_{15}H_{28}$	208	ND	ND	ND	ND	4.03	77	ND	ND

Octanoic acid-tert butyl ester	12.795	$C_{12}H_{24}O_2$	200	ND	ND	ND	ND	ND	ND	2.76	74
8,10-Dioxaheptadecane	12.797	$C_{15}H_{32}O_2$	244	0.86	81	8.76	81	6.26	80	ND	ND
3,4-Dimethylpent-2-en-1-ol	12.820	$C_7H_{14}O$	114	ND	ND	1.90	63	ND	ND	ND	ND
Propanoicacid, 2-methyl-, octyl ester	12.825	$C_{12}H_{24}O_2$	200	ND	ND	ND	ND	ND	ND	2.24	74
Propanoic acid, 2-methyl-, 3-hydroxy-2,2,4-trimethylpentyl ester	12.956	$C_{12}H_{24}O_3$	216	ND	ND	ND	ND	ND	ND	2.52	90
2,6,10-Trimethyltridecane	13.519	$C_{16}H_{34}$	226	0.48	94	ND	ND	ND	ND	ND	ND
1-Tetradecanol	13.596	$C_{14}H_{30}O$	214	ND	ND	ND	ND	ND	ND	2.76	91
Cycloheptasiloxane, tetradecamethyl-	13.738	$C_{14}H_{42}O_7Si_7$	518	1.59	70	7.59	83	6.76	85	4.24	83
Silane, trichlorooctadecyl-	13.770	$C_{18}H_{37}Cl_3Si$	386	ND	ND	4.09	78	ND	ND	ND	ND
Pentadecane	13.772	$C_{15}H_{32}$	212	5.51	96	ND	ND	ND	ND	ND	ND
Nonane, 5-(2-methylpropyl)-	13.774	$C_{13}H_{28}$	184	0.56	83	ND	ND	3.84	87	3.53	90
2,4-Di-tert-butylphenol	13.883	$C_{14}H_{22}O$	206	ND	ND	ND	ND	ND	ND	8.61	96
9-Octadecene, (E)-	13.886	$C_{18}H_{36}$	252	0.60	94	ND	ND	ND	ND	ND	ND
Tetradecane, 4-methyl-	14.073	$C_{15}H_{32}$	212	ND	ND	ND	ND	ND	ND	2.11	90
Pentadecane, 6-methyl-	14.097	$C_{16}H_{34}$	226	0.70	94	ND	ND	ND	ND	ND	ND
Cycloundecane, 1,1,2-trimethyl-	14.164	$C_{14}H_{28}$	196	0.93	92	ND	ND	ND	ND	ND	ND
9-Octadecene, (E)-	14.225	$C_{18}H_{36}$	252	0.87	91	ND	ND	ND	ND	ND	ND
Pentadecafluorooctanoic acid, dodecyl ester	14.315	$C_{20}H_{25}F_{15}O_2$	582	0.59	90	ND	ND	ND	ND	ND	ND
Pentadecane	14.433	$C_{15}H_{32}$	212	4.97	95	ND	ND	ND	ND	ND	ND
Diethyl Phthalate	14.458	$C_{12}H_{14}O_4$	222	ND	ND	12.90	96	25.52	97	35.58	97
(-)-Globulol	14.611	$C_{15}H_{26}O$	222	0.46	93	ND	ND	ND	ND	ND	ND
Cyclooctasiloxane, hexadecamethyl-	14.844	$C_{16}H_{48}O_8Si_8$	592	ND	ND	2.96	85	2.88	83	1.94	85
3-Octadecene, (E)-	14.948	$C_{18}H_{36}$	252	2.33	96	ND	ND	ND	ND	ND	ND
Eicosane, 10-methyl-	15.060	$C_{21}H_{44}$	296	64.98	96	6.73	94	ND	ND	ND	ND
1-Decanol, 2-hexyl-	15.167	$C_{16}H_{34}O$	242	0.56	92	ND	ND	ND	ND	ND	ND
Tridecane, 6-methyl-	15.342	$C_{14}H_{30}$	198	2.74	93	ND	ND	ND	ND	ND	ND
2-Pentadecanone, 6,10,14-trimethyl-	15.929	$C_{18}H_{36}O$	268	1.98	95	ND	ND	ND	ND	ND	ND
Octadecane, 1-(ethenyloxy)-	16.183	$C_{20}H_{40}O$	296	0.39	84	ND	ND	ND	ND	ND	ND
Bromoacetic acid, octadecyl ester	16.370	$C_{20}H_{39}BrO_2$	390	0.41	83	ND	ND	ND	ND	ND	ND
1,19-Eicosadiene	17.126	$C_{20}H_{38}$	278	1.89	95	ND	ND	ND	ND	ND	ND
1-Tricosene	17.236	$C_{23}H_{46}$	322	0.64	95	ND	ND	ND	ND	ND	ND
Phenol, 4,4′-(1-methylethylidene)bis-	17.774	$C_{15}H_{16}O_2$	228	0.68	89	ND	ND	6.53	88	4.34	91

RT=Retention Time, MW=Molecular Weight, ND=Not Detected.

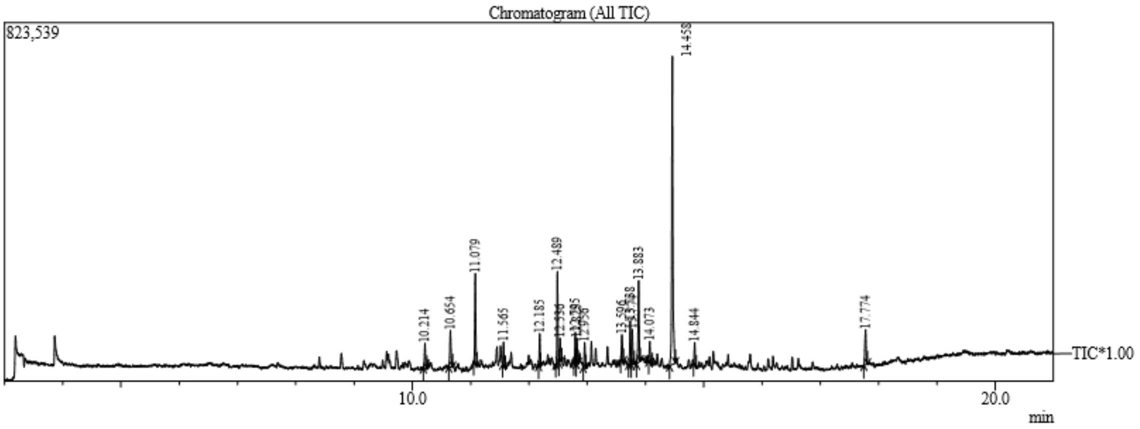

Peak Report TIC

Peak#	R.Time	I.Time	F.Time	Area	Area%	Height	Height%	A/H	Mark	Name
1	10.214	10.190	10.250	69902	2.53	55407	2.55	1.26		Decane, 3,7-dimethyl-
2	10.654	10.625	10.690	109612	3.96	81159	3.73	1.35		Nonanal
3	11.079	11.050	11.110	225846	8.16	201143	9.26	1.12		Cyclopentasiloxane, decamethyl-
4	11.565	11.550	11.600	51987	1.88	51874	2.39	1.00		Decanal
5	12.185	12.165	12.205	75401	2.72	72100	3.32	1.05		Nonane, 5-(2-methylpropyl)-
6	12.489	12.460	12.515	223184	8.06	204250	9.40	1.09		Cyclohexasiloxane, dodecamethyl-
7	12.536	12.515	12.560	56786	2.05	53057	2.44	1.07	V	Nonane, 5-(2-methylpropyl)-
8	12.795	12.770	12.810	76330	2.76	67804	3.12	1.13		Octanoic acid-tert butyl ester
9	12.825	12.810	12.850	62072	2.24	55830	2.57	1.11	V	Propanoic acid, 2-methyl-, octyl ester
10	12.956	12.930	12.980	69707	2.52	56253	2.59	1.24		Propanoic acid, 2-methyl-, 3-hydroxy-2,2,
11	13.596	13.575	13.625	76305	2.76	57645	2.65	1.32		1-Tetradecanol
12	13.738	13.710	13.755	117346	4.24	96032	4.42	1.22		Cycloheptasiloxane, tetradecamethyl-
13	13.774	13.755	13.805	97769	3.53	77493	3.57	1.26	V	Nonane, 5-(2-methylpropyl)-
14	13.883	13.845	13.910	238339	8.61	181225	8.34	1.32		2,4-Di-tert-butylphenol
15	14.073	14.055	14.115	58319	2.11	46594	2.14	1.25		Tetradecane, 4-methyl-
16	14.458	14.415	14.515	984641	35.58	686330	31.58	1.43		Diethyl Phthalate
17	14.844	14.825	14.875	53806	1.94	50713	2.33	1.06		Cyclooctasiloxane, hexadecamethyl-
18	17.774	17.745	17.810	120168	4.34	78231	3.60	1.54		Phenol, 4,4'-(1-methylethylidene)bis-
				2767520	100.00	2173140	100.00			

Figure S1. Chromatogram that shows the results of the analysis of sample of f/2 medium prepared using HS-SPME.

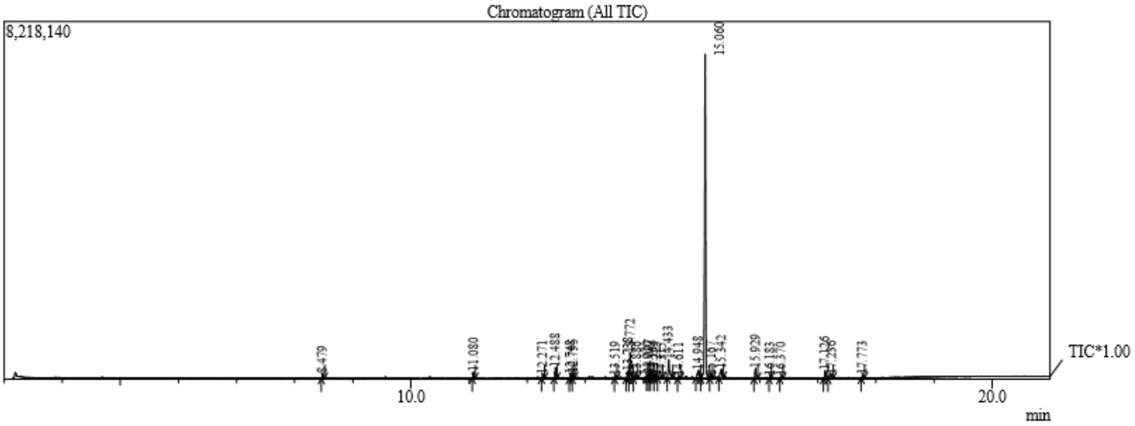

Peak Report TIC

Peak#	R.Time	I.Time	F.Time	Area	Area%	Height	Height%	A/H	Mark	Name
1	8.479	8.460	8.510	109565	0.79	100914	0.93	1.09		Oxime-, methoxy-phenyl-_
2	11.080	11.055	11.105	142683	1.03	139764	1.29	1.02		Cyclopentasiloxane, decamethyl-
3	12.271	12.245	12.300	106787	0.77	86870	0.80	1.23		3-tert-Butyl-2-pyrazolin-5-one
4	12.488	12.460	12.515	261646	1.89	237503	2.20	1.10		Cyclohexasiloxane, dodecamethyl-
5	12.748	12.720	12.775	110567	0.80	92521	0.86	1.20		2,2,6,7-Tetramethyl-10-oxatricyclo[4.3.0.
6	12.795	12.775	12.820	119074	0.86	109172	1.01	1.09		8,10-Dioxaheptadecane
7	13.519	13.500	13.545	66497	0.48	62444	0.58	1.06		2,6,10-Trimethyltridecane
8	13.738	13.700	13.750	219489	1.59	154910	1.43	1.42		Cycloheptasiloxane, tetradecamethyl-
9	13.772	13.750	13.825	762651	5.51	574786	5.31	1.33	V	Pentadecane
10	13.886	13.825	13.905	82975	0.60	59412	0.55	1.40	V	9-Octadecene, (E)-
11	14.070	14.055	14.085	76845	0.56	51582	0.48	1.49		Nonane, 5-methyl-5-propyl-
12	14.097	14.085	14.120	96506	0.70	89318	0.83	1.08	V	Pentadecane, 6-methyl-
13	14.164	14.120	14.180	129221	0.93	66978	0.62	1.93		Cycloundecane, 1,1,2-trimethyl-
14	14.225	14.180	14.245	120983	0.87	50342	0.47	2.40	V	9-Octadecene, (E)-
15	14.315	14.245	14.330	82310	0.59	31012	0.29	2.65	V	Pentadecafluorooctanoic acid, dodecyl est
16	14.433	14.410	14.505	687530	4.97	413712	3.83	1.66		Pentadecane
17	14.611	14.585	14.640	63053	0.46	45878	0.42	1.37		(-)-Globulol
18	14.948	14.900	14.985	322886	2.33	175156	1.62	1.84		3-Octadecene, (E)-
19	15.060	14.985	15.140	8995736	64.98	7406639	68.49	1.21		Eicosane, 10-methyl-
20	15.167	15.140	15.195	77462	0.56	59817	0.55	1.29		1-Decanol, 2-hexyl-
21	15.342	15.300	15.375	379007	2.74	183550	1.70	2.06		Tridecane, 6-methyl-
22	15.929	15.900	15.970	273889	1.98	222897	2.06	1.23		2-Pentadecanone, 6,10,14-trimethyl-
23	16.183	16.160	16.200	54042	0.39	45069	0.42	1.20		Octadecane, 1-(ethenyloxy)-
24	16.370	16.345	16.400	56711	0.41	46035	0.43	1.23		Bromoacetic acid, octadecyl ester
25	17.126	17.095	17.175	261973	1.89	173481	1.60	1.51		1,19-Eicosadiene
26	17.236	17.175	17.265	88715	0.64	68786	0.64	1.29	V	1-Tricosene
27	17.773	17.745	17.805	94748	0.68	66256	0.61	1.43		Phenol, 4,4'-(1-methylethylidene)bis-

Figure S2. Chromatogram that shows the results of the analysis of sample of phenotype Type 1 of *Synechococcus* sp. prepared using HS-SPME.

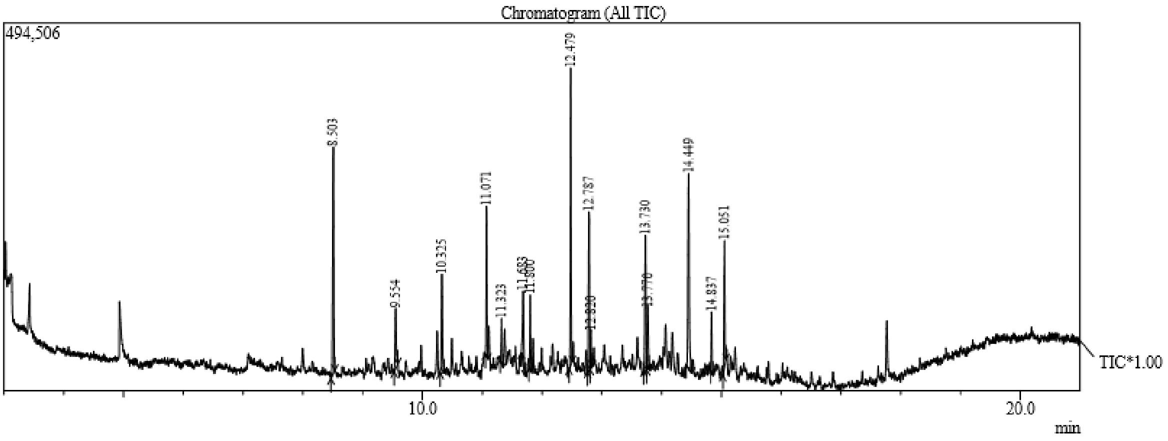

Peak Report TIC

Peak#	R.Time	I.Time	F.Time	Area	Area%	Height	Height%	A/H	Mark	Name
1	8.503	8.470	8.535	274400	12.91	226454	12.66	1.21		Oxime-, methoxy-phenyl-_
2	9.554	9.530	9.585	74777	3.52	59586	3.33	1.25		Cyclotetrasiloxane, octamethyl-
3	10.325	10.295	10.355	117447	5.52	98154	5.49	1.20		Cyclotrisiloxane, hexamethyl-
4	11.071	11.050	11.095	157021	7.39	149491	8.36	1.05		Cyclopentasiloxane, decamethyl-
5	11.323	11.295	11.350	58178	2.74	44555	2.49	1.31		Acetoxyacetic acid, 5-tetradecyl ester
6	11.683	11.655	11.715	98945	4.65	71697	4.01	1.38		Bicyclo[3.1.1]hept-3-en-2-one, 4,6,6-trim
7	11.800	11.780	11.825	77750	3.66	74115	4.14	1.05		Artedouglasia oxide A
8	12.479	12.450	12.510	311915	14.67	295893	16.54	1.05		Cyclohexasiloxane, dodecamethyl-
9	12.787	12.760	12.805	186198	8.76	157707	8.82	1.18		8,10-Dioxaheptadecane
10	12.820	12.805	12.840	40488	1.90	36890	2.06	1.10	V	3,4-Dimethylpent-2-en-1-ol
11	13.730	13.700	13.750	161416	7.59	134128	7.50	1.20		Cycloheptasiloxane, tetradecamethyl-
12	13.770	13.750	13.795	86950	4.09	60397	3.38	1.44	V	Silane, trichlorooctadecyl-
13	14.449	14.415	14.500	274337	12.90	193430	10.82	1.42		Diethyl Phthalate
14	14.837	14.820	14.860	62992	2.96	59963	3.35	1.05		Cyclooctasiloxane, hexadecamethyl-
15	15.051	15.025	15.075	143095	6.73	125993	7.04	1.14		Eicosane, 10-methyl-
				2125909	100.00	1788453	100.00			

Figure S3. Chromatogram that shows the results of the analysis of sample of phenotype Type 2 of *Synechococcus* sp. prepared using HS-SPME.

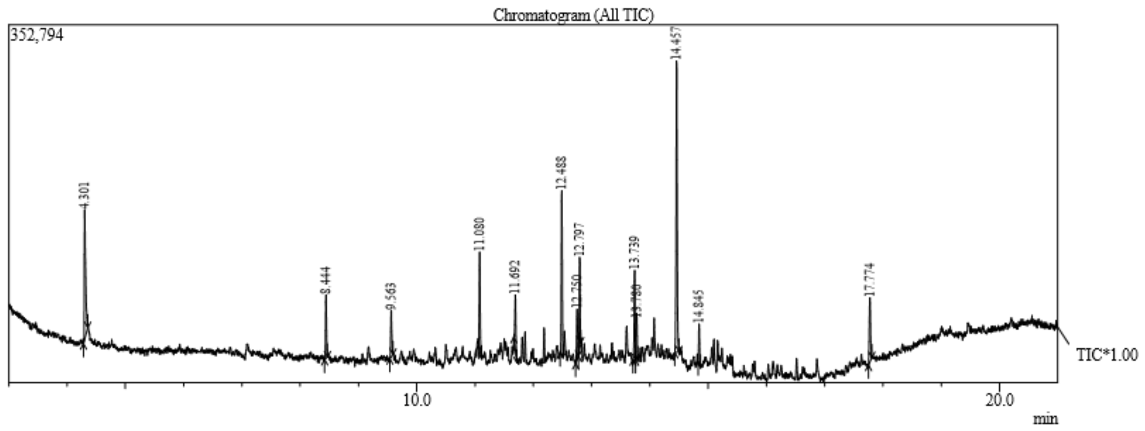

Peak Report TIC

Peak#	R.Time	I.Time	F.Time	Area	Area%	Height	Height%	A/H	Mark	Name
1	4.301	4.280	4.355	180520	13.03	115843	10.57	1.56		Silanediol, dimethyl-
2	8.444	8.420	8.470	62420	4.51	56571	5.16	1.10		Oxime-, methoxy-phenyl-_
3	9.563	9.540	9.590	48911	3.53	41209	3.76	1.19		trisiloxane, 1,1,1,5,5,5-hexamethyl-3-[(tri
4	11.080	11.060	11.115	93593	6.75	87709	8.01	1.07		Cyclopentasiloxane, decamethyl-
5	11.692	11.665	11.720	55252	3.99	48764	4.45	1.13		Bicyclo[3.1.1]hept-3-en-2-one, 4,6,6-trim
6	12.488	12.460	12.515	171635	12.39	147370	13.45	1.16		Cyclohexasiloxane, dodecamethyl-
7	12.750	12.725	12.775	55771	4.03	45121	4.12	1.24		Naphthalene, decahydro-1,4a-dimethyl-7-
8	12.797	12.775	12.815	86707	6.26	81979	7.48	1.06		8,10-Dioxaheptadecane
9	13.739	13.710	13.755	93707	6.76	80077	7.31	1.17		Cycloheptasiloxane, tetradecamethyl-
10	13.780	13.755	13.795	53196	3.84	36340	3.32	1.46	V	Nonane, 5-(2-methylpropyl)-
11	14.457	14.425	14.500	353563	25.52	260864	23.81	1.36		Diethyl Phthalate
12	14.845	14.830	14.870	39854	2.88	36108	3.30	1.10		Cyclooctasiloxane, hexadecamethyl-
13	17.774	17.750	17.810	90417	6.53	57570	5.26	1.57		Phenol, 4,4'-(1-methylethylidene)bis-
				1385546	100.00	1095525	100.00			

Figure S4. Chromatogram that shows the results of the analysis of sample of phenotype Type 3a of *Synechococcus* sp. prepared using HS-SPME.
